# Automated Assembly of Large-Scale Aerospace Components: A Structured Narrative Survey of Emerging Technologies

**DOI:** 10.3390/s26082294

**Published:** 2026-04-08

**Authors:** Kuai Zhou, Wenmin Chu, Peng Zhao, Xiaoxu Ji, Lulu Huang

**Affiliations:** 1 School of Aeronautical Engineering, Nanjing University of Industry Technology, Nanjing 210023, China; 2024101500@niit.edu.cn (K.Z.);; 2 College of Mechanical and Electrical Engineering, Nanjing University of Aeronautics and Astronautics, Nanjing 210016, China; 3 School of Mechanical and Automotive Engineering, West Anhui University, Lu’an 237012, China

**Keywords:** large-scale components, automated assembly, pose adjustment and positioning, digital measurement, trajectory planning, survey

## Abstract

Large-scale aerospace components (e.g., wings, fuselage sections, wing boxes, and rocket segments) feature large dimensions, low stiffness, complex interfaces, and strict assembly tolerances. Traditional rigid tooling and manual alignment struggle to meet the demands of high precision, efficiency, and flexibility in modern aerospace manufacturing. This paper presents a structured literature review on the automated assembly of large-scale aerospace components, summarizing advances in three core domains: pose adjustment and positioning mechanisms, digital measurement technologies, and trajectory planning and control. Particular emphasis is placed on two cross-cutting themes: measurement uncertainty analysis and flexible assembly, which are critical for high-quality docking. The review classifies pose adjustment mechanisms into four categories (NC positioners, parallel kinematic machines, industrial robots, and novel mechanisms) and digital measurement into five branches (vision metrology, large-scale metrology, measurement field construction, uncertainty analysis, and auxiliary techniques). It also outlines five trajectory planning and control routes, covering traditional methods, multi-sensor fusion, digital twins, flexible assembly, and emerging intelligent approaches. The analysis reveals that current research suffers from fragmentation among mechanism design, metrology, and control, with insufficient integration of uncertainty propagation and flexible deformation modeling. Future systems will rely on heterogeneous equipment collaboration, uncertainty-aware closed-loop control, high-fidelity flexible modeling, and digital twin-driven decision-making. This review provides a unified framework and a technical reference for developing reliable, flexible, and scalable automated assembly systems for next-generation aerospace structures.

## 1. Literature Retrieval and Screening Methodology

This paper adopts a structured literature review approach to summarize representative research in the field of automated assembly of large-scale aerospace components, focusing on three core directions: pose adjustment and positioning mechanisms, digital measurement technologies, and trajectory planning and control. Particular attention is paid to two rapidly developing research topics in recent years: measurement uncertainty analysis and flexible assembly. Based on current retrieval records, a total of 231 highly relevant studies have been collected, with an estimated coverage of approximately 91% for related topics. Therefore, the methodology section of this paper only reports traceable retrieval and screening information within the current project, and no inferential supplementation is made for intermediate statistics that were not preserved [1,2,3,4].

### 1.1. Retrieval Databases and Time Scope

The retrieval databases include Web of Science Core Collection, Scopus, IEEE Xplore, and CNKI. The time scope focuses on the literature from the past 10 to 15 years, with a key emphasis on studies since 2008, while retaining a small number of early foundational studies on measurement-assisted assembly, reconfigurable tooling, and flexible assembly to ensure the integrity of the technological evolution context [2,4,5,6].

### 1.2. Literature Inclusion Criteria

The literature included in this review must satisfy all of the following criteria:

- The research object is directly related to the automated assembly of large-scale aerospace components, with typical objects including wings, fuselage sections, wing boxes, cabin sections, wall panels, and large-scale composite components.
- The research topic falls within the core technical domains focused on in this paper, namely pose adjustment and positioning mechanisms, digital measurement technologies, measurement field construction and uncertainty analysis, trajectory planning and control, flexible assembly, or digital twin-assisted assembly.
- The literature types are mainly peer-reviewed journal papers and formal conference papers, with appropriate inclusion of dissertations or research reports with clear academic review mechanisms and explicit methodological contributions.
- The literature can provide a clear methodological framework, system structure, experimental verification, industrial cases, or review-based induction, rather than only conceptual description.
- The content of the literature can directly support the key issues discussed in this paper, especially studies involving measurement-assisted assembly, measurement uncertainty propagation, flexible component shape control, assembly gap control, and digital twin closed-loop control [7,8,9,10,11,12].

### 1.3. Literature Exclusion Criteria

The following types of the literature are not included in the main text analysis:

- Studies weakly associated with the assembly of large-scale aerospace components, only discussing general robotic assembly, ordinary mechanical assembly, or non-large-scale manufacturing scenarios.
- Non-academic materials such as product promotions, enterprise samples, web introductions, press releases, or materials lacking complete technical details.
- Abstracts, short communications, briefs, or informal materials with high duplication with the topic of this paper but limited incremental technical information.
- Literature with unconfirmed source reliability or lacking support from methods, experiments, and results.
- Materials that only paraphrase standard content without providing engineering applications, experimental verifications, or methodological comparisons.

### 1.4. Literature Screening and Classification Process

In the current project workflow, the retrieval task first generates a highly relevant candidate literature set based on research objectives, followed by secondary screening and structured classification around the review topic. Candidate studies are judged mainly by whether they can support the three core technical lines of this paper: pose adjustment and positioning mechanisms, digital measurement technologies, and trajectory planning and control. The included studies are further classified and sorted according to technical routes, application objects, verification levels, and whether they involve uncertainty or flexibility issues. To reduce literature bias caused by research regions and research groups, the screening process focuses on representative achievements of Airbus, Boeing, and related universities and research institutions, and strives to maintain balanced coverage of research results from China, Europe, and North America [1,2,13,14,15].

## 2. Introduction

Large-scale aerospace components, such as wings, fuselage sections, wing boxes, cabin sections, and rocket barrel sections, are generally characterized by large dimensions, low relative stiffness, complex docking interfaces, and strict assembly tolerance requirements. In actual assembly processes, geometric deviations are not only determined by the nominal dimensions of parts, but jointly formed by part manufacturing fluctuations, tooling errors, gravity and temperature deformations, measurement uncertainty, and assembly control errors [1,2,7]. With the continuous development of aerospace products toward large-scale integrated structures, high composite material ratios, higher quality requirements, and more flexible production modes, traditional heavy-duty tooling and highly manual alignment assembly methods are increasingly difficult to simultaneously meet multiple requirements such as precision, efficiency, adaptability, and cost [3,15,16]. Against this background, automated assembly of large-scale aerospace components has become a key supporting direction in the manufacturing of new-generation aircraft [2,5].

An essential feature of this field is its strong system coupling. The performance of automated docking assembly of large-scale components does not depend on a single technology, but is jointly determined by pose adjustment and positioning mechanisms, large-scale digital measurement, trajectory planning and control, and the increasingly important digital and data-driven infrastructure [1,2,15]. In this sense, the problem is not merely “how to move a large component to a target position”, but “how to generate, perceive, predict, and control the geometric state of a flexible assembly system throughout the entire docking process” [7,16]. This system perspective is particularly important because the independent improvement of a certain technical link (e.g., higher measurement accuracy or higher actuator accuracy) will not automatically translate into better assembly quality unless it is effectively integrated with the entire assembly process chain [2,5].

Although an increasing number of review studies have emerged in recent years around reconfigurable tooling, measurement-assisted assembly, large-scale metrology, and assembly deviation modeling, existing reviews mostly focus on a single technical slice of the problem and rarely conduct a systematic review from the complete process chain of automated assembly of large-scale components [1,3,4,7]. Reviews on tooling and fixtures usually emphasize reconfigurability and automated equipment [3], reviews on metrology focus more on measurement principles and instrument capabilities [4], and reviews on aircraft assembly deviations mainly focus on geometric deviation propagation with less coverage of integrated control issues in docking assembly [7]. Therefore, three obvious gaps remain in this field:

1. A unified review framework that connects pose adjustment and positioning mechanisms, digital measurement, and planning and control is lacking.
2. The rapidly developing contents in recent years, such as measurement uncertainty analysis, digital twin-assisted decision-making, and flexible assembly of large flexible composite structures, have not been fully integrated within the scope of a single review [2,16].
3. The existing research landscape is still fragmented among the three major research groups in China, Europe, and North America, while many key advances in this field are precisely driven by the joint aerospace manufacturing practices of these regions [1,2,4].

To address the above gaps, this paper systematically reviews the emerging technologies in the automated assembly of large-scale aerospace components. The research objects of this paper cover both large-scale structural components in the aviation and aerospace fields, including but not limited to aircraft wing and fuselage assembly, wing-box structures, large wall panels, aerospace barrel sections, and other large docking components. The review time scope focuses on the past 10 to 15 years, while retaining a small number of foundational early work to illustrate the formation context of main technical routes. Instead of treating assembly as an isolated robotic motion problem, this paper systematically combs the key technical system of automated assembly of large-scale components covering the complete technical chain from mechanisms and measurement to planning and control.

Based on this goal, relevant studies are summarized into three main technical domains:

1. Pose adjustment and positioning mechanisms for docking assembly of large-scale components, including Numerical Control (NC) positioners, PKMs, industrial robots, and other novel mechanisms.
2. Digital measurement technologies for docking assembly of large-scale components, including vision measurement, large-scale measurement equipment, measurement field construction, and measurement uncertainty analysis.
3. Trajectory planning and control for docking assembly of large-scale components, including traditional planning and control methods, multi-sensor fusion, digital twin technology, flexible assembly technology, and other emerging intelligent methods.

This three-part classification framework is adopted because it well corresponds to the execution layer, perception layer, and decision layer in the automated assembly system of large-scale components, while retaining the close coupling relationship among the three [2,5,16].

Under this overall framework, this paper particularly emphasizes two cross-cutting themes:

1. Measurement uncertainty analysis: The assembly quality of large-scale components depends not only on “what is measured”, but also on whether the uncertainty of measurement results can be strictly quantified and further transmitted to pose adjustment, docking, and tolerance judgment [4,8].
2. Flexible assembly technology: With the wide application of large thin-walled parts and composite structures, the traditional rigid body assumption is increasingly difficult to meet the planning and quality control requirements in the docking assembly of large-scale components [1,7].

These two themes are not regarded as isolated issues in this paper, but as key horizontal threads that redefine the design and evaluation methods of automated assembly systems.

**Figure 1:**
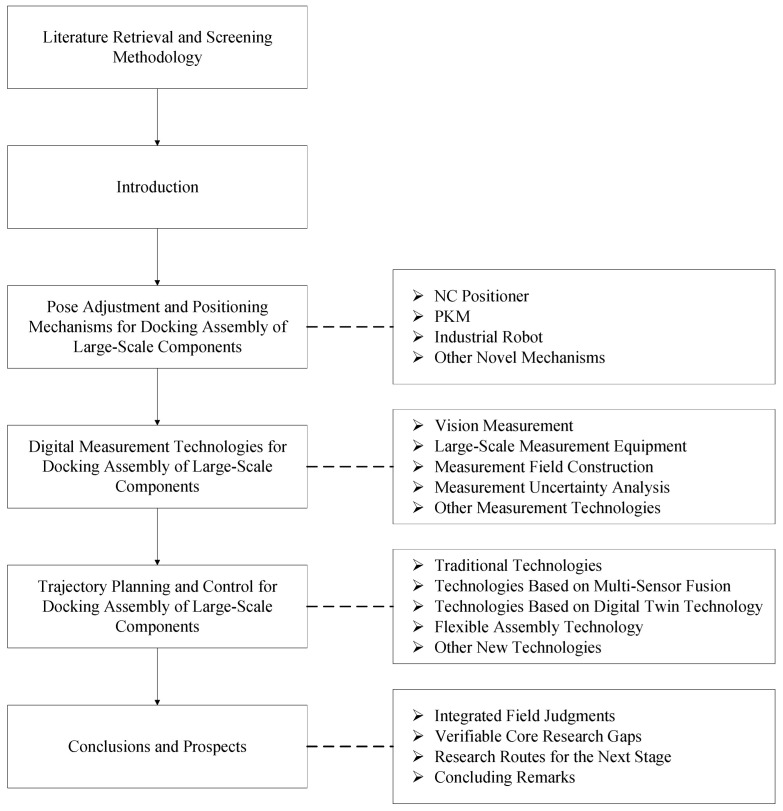
Systematic context.

The rest of this paper is organized as follows: Section 3 reviews pose adjustment and positioning mechanisms for docking assembly of large-scale components. Section 4 reviews digital measurement technologies for docking assembly of large-scale components, focusing on large-scale metrology, measurement field construction, and measurement uncertainty analysis. Section 5 reviews trajectory planning and control methods for docking assembly of large-scale components, including multi-sensor-driven, digital twin-assisted, and flexible assembly technical routes. Through this structure, this paper aims to establish a clear and coherent research framework for this field, sort out the relationships between main technical routes, and provide a systematic reference for the future development of high-precision and high-flexibility automated assembly systems for large-scale aerospace components. The systematic context of this paper is shown in Figure 1.

## 3. Pose Adjustment and Positioning Mechanisms for Docking Assembly of Large-Scale Components

The difficulty of docking assembly for large-scale aerospace components lies not only in the large size of the components, but also in the fact that their geometric accuracy is not determined by a single part size, but dynamically generated under the combined action of thin-walled part deformation, tooling errors, measurement errors, and assembly loads. Traditional heavy-duty rigid tooling maintains assembly relationships through high stiffness and geometric replication, but its long design and manufacturing cycle, high reconfiguration cost, and slow response to model changes make it difficult to adapt to the development needs of composite structures, small-batch and multi-model production, and digital assembly [1,2,3]. Therefore, the technical evolution main line of pose adjustment and positioning mechanisms is essentially shifting the source of precision from fixed master tooling to reconfigurable mechanisms, online metrology, and closed-loop compensation systems [2,3,5].

From existing research and engineering practice, pose adjustment and positioning mechanisms for docking assembly of large-scale components can be classified into four categories:

1. NC positioners and reconfigurable fixtures: They are characterized by high load-bearing capacity and high compatibility with existing process chains.
2. PKMs: The core value lies in providing multi-Degree of Freedom (DOF) pose adjustment and high structural stiffness under relatively high load-bearing conditions.
3. Industrial robots: They have outstanding features of large workspace, strong versatility, and relatively controllable cost, but require calibration, external measurement, and special end-effectors to enter high-precision aviation assembly scenarios.
4. Novel mechanisms for low-accessibility, mobile, and fixed-station-free assembly: The main role is to expand the assembly space that is difficult to cover by traditional tooling and robots [1,15,17].

Table 1 provides a comprehensive comparison of the four types of pose adjustment and positioning mechanisms in terms of load-bearing capacity, workspace, representative positioning accuracy, stiffness, cost, typical applications, and technological maturity. It should be noted that the positioning accuracy indicators in the literature are usually strongly task-related, and different studies adopt evaluation objects including absolute positioning of support points, hole position errors, surface positioning accuracy of parts, and pose alignment accuracy, so they cannot be simply regarded as fully comparable unified indicators. Therefore, the “Positioning Accuracy” column in the table prioritizes representative results that can be directly cited; when unified numerical values are lacking, task-related descriptions are retained.

It should be noted that the four evaluations (load-bearing capacity, workspace, stiffness, and cost) in Table 1 adopt relative grading rather than absolute numerical grading. Their judgments are based on the typical engineering performance of various mechanisms in the docking assembly scenarios of large-scale components, rather than the extreme parameters of a single model of equipment.

Among them, load-bearing capacity is mainly classified according to the mechanism’s ability to withstand the self-weight of large-scale components, process loads, and contact loads under the premise of ensuring assembly stability: those capable of stably bearing the main loads of support and docking of large-scale components for a long time are classified as “High”; those only able to bear medium-sized components or requiring auxiliary support are classified as “Medium”; and those mainly applicable to local operations, light-load tasks, or end compensation are classified as “Low”.

Workspace is mainly divided based on the assembly area that the mechanism can effectively cover and its adaptability to the pose adjustment of large-sized components: those that can cover a wide assembly area or have significant spatial extensibility are classified as “Large”; those suitable for typical stations but limited by topology or layout are classified as “Medium”; and those only applicable to local areas, narrow spaces, or operations near specific poses are classified as “Small”.

Stiffness is mainly graded according to the mechanism’s ability to maintain pose stability, suppress elastic deformation, and avoid error amplification under assembly forces, contact adjustments, and process disturbances: those that can stably maintain posture under high loads and contact conditions are classified as “High”; those that can work under general assembly loads but still have a significant dependence on external compensation are classified as “Medium”; and those prone to structural compliance impacts under process loads or large-range operations are classified as “Low”.

Cost is relatively evaluated from a full-life-cycle perspective, considering not only equipment procurement costs but also system integration, on-site deployment, calibration and maintenance, and subsequent reconfiguration costs: those with high initial investment and integration complexity are classified as “High”; those with mature hardware and relatively controllable expansion costs are classified as “Medium”; and those with relatively low equipment body and application thresholds are classified as “Low”.

Therefore, the significance of the grades in the table is to support horizontal comparison between different technical routes, rather than providing fixed numerical conclusions divorced from specific task conditions.

### 3.1. NC Positioner

NC positioners are the earliest type of mechanisms to achieve engineering upgrading in the assembly of large-scale components. The key transformation lies not in simply adding servo axes to traditional jig frames, but in changing the way tooling precision is generated. The deterministic tooling concept proposed by Hartmann et al. for Airbus wing assembly shows that under the support of 3D digital prototypes and high-precision NC manufacturing, the geometric relationships originally maintained by large welded structures for a long time can be transferred to modular datum interfaces and tool units that are manufacturable, replaceable, and verifiable [13]. The core innovation is to transform one-time formed heavy-duty master tooling into a tool system defined by digital models, assembled according to datums and developed in parallel with products. This route has been proven to support the parallel design and manufacturing of large wing assembly tooling in the A380 and A340-600 wing (Airbus, Broughton, UK)assembly projects, thereby improving the ability to absorb design changes and shortening the tooling preparation cycle [13].

On this basis, the Affordable Reconfigurable Tooling (ART) proposed by Kihlman and Engström further advances NC positioners from “manufacturable digital tooling” to “reconfigurable digital tooling” [18]. The real innovation of ART is not only the disassembly of fixtures, but also treating tooling reconfiguration itself as part of automated tasks. The study proposes a tooling system composed of modular support units and reconfigurable skeletons, enabling the same platform to switch quickly between products of the same series and continue to be reused by reconstructing local units when product families change [18]. This transforms tooling assets from exclusive resources for a single model to shared resources for multiple projects, which is particularly suitable for scenarios with frequent prototype, low-volume models, and product iterations.

The Coordinate Controlled Fixturing proposed by Jonsson and Ossbahr further changes the construction logic of large-scale tooling [20]. This method no longer pursues the overall replication of all nominal geometries of the workpiece, but only performs high-precision online configuration of key support points in direct contact with parts. Relying on external metrology systems, the BoxJoint modular frame and Flexapod support unit can complete configuration adjustments on the workshop site [20]. The core innovation of this method is to convert the offline manufacturing accuracy of traditional tooling into online generated accuracy relying on external metrology systems. Thus, the high-precision requirements in the assembly of large-scale components no longer need to be fully realized through super-large rigid steel structures, but can be achieved through high-precision online reconfiguration of a small number of key support points. This not only reduces tooling manufacturing and recertification costs, but also provides a realistic basis for flexible assembly [20].

The Flexapods industrial case in the Saab nEURON project by Kihlman and Engström provides direct evidence that reconfigurable NC positioners have entered real aviation scenarios [21]. This system combines Flexapod with a laser tracker in a closed loop and guides manual fine-tuning of each joint of the mechanism in real time through operating software, finally achieving an absolute positioning accuracy of approximately ±0.05 mm for support points [21]. The significance of this result lies not only in the numerical value itself, but also in proving that reconfigurable support units have the on-site accuracy generation capability required for prototype-level aviation assembly. Compared with traditional welded steel structure tooling, Flexapods show obvious advantages in scenarios with frequent design changes and high tooling reuse requirements [21].

For composite wing-box assembly, the role of NC positioners has further expanded from “support and positioning” to “active realization of key characteristics” tools. Millar et al. discussed the universal value of reconfigurable flexible tooling in wing assembly, pointing out its great significance for reducing the cost of special tooling and shortening deployment cycles [37]. Bakker et al. went further, directly conducting research on rib hole key characteristics in composite wing-box assembly, and proposed a tooling and fixture system with adjustable datum pickup capability [22]. This scheme does not passively accept part forming errors, but actively absorbs part deviations through adjustable supports and datum reconstruction to bring key hole systems back to an acceptable range. A special test bench was built in the paper, and the ability of this type of reconfigurable fixture to realize key characteristics was verified through experiments [22]. This indicates that NC positioners are no longer just tools for “maintaining geometric relationships”, but tools for “actively redistributing errors”.

The META live fixturing proposed by Maropoulos’s team marks the development of NC positioners towards the “measurement-enhanced tooling” stage [19]. In this wing-box case, the tooling is no longer a passive object for periodic manual verification, but an active system that is continuously observable during the assembly process through embedded metrology and online update mechanisms [19]. The core innovation is to use on-site measurement to support tooling state correction, reducing the need to rely on large-volume single steel structures to maintain accuracy for a long time [19]. Vaughan et al.’s research on adaptive fixtures further indicates that the future development direction of NC positioners is not simply to improve structural stiffness, but to enhance their response ability to part differences and process disturbances [38].

Overall, the greatest contribution of the NC positioner route is to shift the assembly of large-scale components from “replicating geometry relying on static masters” to “generating geometry relying on key datums, adjustable supports, and online metrology” [2,19]. Its advantages include high load-bearing capacity, high process compatibility, easy integration with traditional assembly processes, and good reconfiguration value in prototype and multi-model environments [18,21]. Its limitation is that DOFs are usually concentrated on limited support nodes, and its flexibility is still inferior to PKMs and robotic systems in continuous large-range pose adjustment and high-frequency dynamic compensation [1,3].

Synthesizing existing research reveals that the development focus of NC positioners lies not merely in increasing adjustable DOF, but in transforming the way assembly precision is generated. From deterministic tooling [13] to ART and coordinate-controlled fixturing [18,20], and further to Flexapod and measurement-enhanced tooling [19,21], the common trend is shifting the precision originally maintained by integral rigid master tooling to precision co-generated by key support points, local adjustable units, and external measurement closed loops. In other words, the true universal principle formed by this category of mechanisms is not full-scale high-precision replication, but high-precision control centered on key characteristics.

Further analysis shows that this technical route actually addresses both stiffness and flexibility requirements through system stratification. Large-scale frameworks are primarily responsible for load-bearing and stability, while local adjustment units and datum interfaces handle pose correction and precision generation [13,20,21]. Therefore, the so-called unification of “high stiffness” and “high flexibility” mentioned in relevant literature is not naturally achieved simultaneously by the same structure, but through the functional separation of load-bearing paths and precision paths. This is also the fundamental reason why NC positioners can balance engineering feasibility and a certain degree of reconfiguration capability.

However, the limitations of existing research are equally evident. Most studies verify support point positioning accuracy, tooling reconfiguration capability, or key hole characteristic realization capability [21,22], but discussions on how these local precisions are further translated into system-level assembly quality—especially their impacts on interface gaps, assembly internal stresses, and subsequent hole-making consistency—remain insufficient [19,38]. Thus, the NC positioner route has well addressed “how local precision is generated” but has not fully answered “how local precision is stably translated into overall assembly quality”.

### 3.2. PKM

The entry of PKMs into the field of large-scale component assembly stems from a long-standing basic contradiction: the docking of large-scale components requires both high load-bearing capacity and structural stiffness, as well as multi-DOF pose adjustment capabilities, while traditional serial robots are usually difficult to meet aviation assembly requirements in both aspects [3,24]. PKMs form a closed-loop load-bearing path through multiple branches, distributing driving forces and external loads among multiple branches, thus usually providing higher stiffness, better error averaging characteristics, and more stable pose retention capabilities at the same scale [23,24]. This makes them an important candidate solution for the docking of large-scale components such as wings, fuselage sections, and wing boxes.

The research by Shang et al. based on Exechon is a representative work in this direction [23]. This study does not simply move general parallel mechanisms to aviation assembly scenarios, but redefines mechanism requirements around the wing assembly task itself, enabling the platform to achieve multi-DOF positioning while maintaining high structural stability under drilling and riveting loads [23]. The core innovation is to closely couple mechanism topology design with specific assembly processes, so that mechanism performance is not only measured by kinematic indicators, but also jointly determined by load-bearing, stiffness, accessibility, and process reachability. For large-scale wing assembly, this is crucial because the assembly platform must remain stable under the simultaneous action of three types of loads: support, positioning, and process loading.

The novel parallel kinematic mechanism and kinematic design method proposed by Jin et al. for aviation wing assembly reflect the “task-oriented customization” advantage of PKMs [26]. The main innovation is not to follow general machine tools or general six-DOF platforms, but to conduct reverse design for the workspace, accessible poses, stiffness requirements, and process paths around wing components [26]. This makes the resulting mechanism not an abstract high-DOF platform, but a dedicated parallel system optimized for aviation assembly boundary conditions. Subsequently, Jin et al. further expanded the mechanism into a more complete aviation automated assembly platform, indicating that parallel kinematic mechanisms can not only undertake positioning functions, but also form an integrated process unit with high-precision machining and in situ metrology [24,39].

An important contribution of Jin et al. in subsequent work is to emphasize the engineering integrity of PKM systems [24]. The solution includes not only the PKM body, but also supporting links such as lightweight support frames, large workspace transfer methods, dust removal systems, tool selection, and in situ metrology support [24]. This indicates that the application of PKMs in large-scale component assembly is not simply a single mechanism replacing fixtures, but requires the formation of a system solution integrating docking, machining, and inspection. The experimental results in the paper show that the proposed integrated PKM solution successfully supports high-precision large-scale aviation structural machining tasks [24]. Although no unified absolute positioning index for horizontal comparison with other studies is given in the paper, the verification has shown that parallel mechanisms have the ability to enter the real engineering chain.

The self-adapting parallel kinematic machines proposed by Neumann et al. further integrate the high stiffness of parallel platforms with assembly adaptability [40]. The key idea of this study is to use the multi-DOF and high load-bearing characteristics of parallel platforms to absorb local manufacturing deviations in the assembly of large-scale wings and fuselage components, rather than transferring all accuracy requirements upstream to parts [40]. Löchte et al. also pointed out that for assembly tasks of section components such as fuselage sections, specially designed parallel topologies are expected to provide more stable error distribution and higher pose adjustment capabilities than traditional devices [27]. The value of this technical route lies in embedding “error compensation capability” directly into the positioning mechanism body, rather than relying entirely on external additional compensation systems.

Hexapod is one of the most typical engineering implementations of PKMs in large-scale component assembly. Erdem et al. developed a synchronous hexapod platform in the LOCOMACHS project for the docking of wing-box front spars and upper covers, while another hexapod platform with force feedback was used for rib installation [25]. The innovations of this study are mainly reflected in three aspects:

1. The mechanism adopts parametric mechanical design, enabling similar platforms to adapt to different components and working conditions [25].
2. A multi-hexapod synchronous control scheme is proposed, enabling multiple support points to cooperatively manipulate large-scale components instead of adjusting independently [25].
3. Torque sensors are integrated into discrete-state assembly control, enabling the platform to adjust poses according to force feedback during contact, thus undertaking real assembly process control rather than only static positioning [25].

Experiments show that the system can maintain stable positioning of the front spar in the presence of external automatic drilling loads and complete force feedback-based rib installation demonstrations [25]. This indicates that PKMs have evolved from “high-stiffness support platforms” to “active assembly mechanisms that are perceivable, collaborative, and resistant to process disturbances”.

Overall, the PKM route solves the balance between “large-DOF pose adjustment” and “high load-bearing and high stiffness” [24,25]. Compared with NC positioners, it has more advantages in continuous pose adjustment, closed-loop docking, and multi-process collaboration. Compared with industrial robots, it is easier to maintain stable accuracy under assembly-level loads [23,24]. Its disadvantages are that the workspace is usually limited by mechanism topology and singular configurations, system design and offline planning are complex, and on-site deployment costs are relatively high [3,27]. Therefore, PKMs are more suitable for undertaking key docking and support nodes in the assembly of large-scale components, rather than being the only equipment for the entire process.

Studies on PKM have shown that their core value lies not merely in providing multi-DOF pose adjustment capability, but more importantly in maintaining more stable load paths and higher equivalent stiffness during high-load assembly tasks through closed-loop structures [23,24,25]. Therefore, the significance of PKM in large-scale component assembly is not to simply replace general positioning devices, but to attempt unified handling of support, pose adjustment, and process force maintenance within the same mechanism framework [26,40]. Compared with NC positioners, it places greater emphasis on suppressing error amplification effects through intrinsic mechanism characteristics.

However, cross-study comparisons reveal that the discussions on “high stiffness” and “high precision” in PKM literature are often stronger than their horizontally comparable evidence. Most existing achievements are verified based on specific prototypes, specific workspaces, and specific task conditions [23,25,26], while issues such as limited workspace, difficulty in singularity avoidance, and complex system deployment have repeatedly emerged in multiple studies [3,27]. This indicates that the advantage of PKM is more accurately described as being easier to maintain stiffness-dominated stable precision in constrained but high-load key stations, rather than being generally assumed to be superior to other schemes in all scenarios.

In addition, the PKM technical route has revealed an important characteristic: its performance is highly dependent on task-specific customization. Relevant studies generally do not directly migrate general-purpose platforms to assembly scenarios, but infer mechanism topology based on the boundary conditions of wing segment, wing-box, or fuselage docking tasks [26,27]. This enables it to demonstrate strong advantages in specific tasks, but also means its versatility and cross-station reusability are often limited. Therefore, the engineering competitiveness of the PKM route essentially depends on whether high-performance benefits can offset the system complexity costs brought by high customization.

### 3.3. Industrial Robot

The application of industrial robots in the automated assembly of large-scale aerospace components has the strongest industrial appeal due to their mature hardware platforms, large workspaces, strong scalability, and system costs usually lower than large-scale special assembly equipment [15,32]. However, industrial robots inherently have two prominent shortcomings: insufficient absolute positioning accuracy and limited structural stiffness, making it difficult for them to directly meet aviation hole-making, riveting, and high-precision docking requirements [30,32]. Therefore, the real technical core of the industrial robot route is not “replacing special equipment with general robots”, but upgrading general robots into assembly process platforms that can meet task accuracy requirements through special end-effectors, error modeling, external measurement, and closed-loop compensation.

The ONCE one-sided drilling system proposed by DeVlieg et al. is a landmark achievement for industrial robots to enter high-precision aviation machining [28]. The core innovation of this system is not the invention of a new robot, but the integration of mass-produced high-load industrial robots with servo-controlled multi-functional end-effectors into a one-sided drilling platform, placing higher precision requirements on the end process head and error compensation chain [28]. In the skin and skeleton connection task of the trailing edge flap of the F/A-18E/F Super Hornet wing (The Boeing Company, St. Louis, MO, USA), the system enables hole positions to meet the ±0.060 specification requirement, while the worst fluctuation of countersink depth is approximately 0.0025 in [28]. This result indicates that with the support of special end-effectors and compensation strategies, industrial robots can already undertake aviation hole-making tasks traditionally completed by special machine tools.

Applied Accurate Robotic Drilling extends the application of industrial robots to curved fuselage structures [29]. This study improves the positioning accuracy on parts to better than ±0.25 mm by arranging secondary encoders at the output end of each robot axis and establishing an enhanced kinematic model [29]. The real innovation is to convert joint transmission errors that are difficult to directly observe in traditional robot control into error terms that can be compensated through output-end measurement and model correction. The study also verified the feasibility of dual-spindle parallel operation on a dual-opposed system and a full-scale fuselage simulation part [29]. This means that industrial robots not only have the potential to replace labor with a single machine, but also have the ability to form high-cycle parallel automated units.

The proposal of measurement-assisted robotic processing provides a more universal path for the engineering application of industrial robots in the aviation field [32]. Jayaweera and Webb pointed out that robotic machining on large-scale aviation structures must adopt the measurement-assisted mode with external metrology participation to achieve stable quality levels [32]. The research by Wang et al. pushed this concept to the real-time control level. They used a single three-DOF laser tracker to observe robot trajectory errors online and compensate path deviations in real time during drilling and cutting processes [30]. The core innovation of this scheme is to transform the laser tracker from an offline calibration device into an online control sensor, enabling the robot to continuously correct its own errors during execution [30]. Experiments in the paper show that after real-time compensation, the trajectory accuracy, hole position accuracy, hole quality, and aluminum part machining accuracy of the robot are significantly improved [30]. Although no unified single absolute error value is given in the abstract of the paper, the results clearly show that external metrology closed-loop can significantly improve the usability of industrial robots in aviation machining.

Multi-sensor collaboration further expands the accuracy boundary of industrial robots. The dual-machine drilling and riveting unit developed by Mei et al. has been systematically designed from structural design, frame chain modeling to multi-sensor positioning methods [31]. The innovation lies in that different sensors undertake the correction of different types of errors. Laser displacement sensors are responsible for aligning the drilling axis direction, and monocular vision is responsible for reference hole recognition and tool center point correction, enabling hierarchical processing of direction errors and position errors [31]. In this automatic drilling and riveting unit, after task space calibration, pose alignment unit calibration, and implicit hand-eye relationship drift calibration, the system finally achieves a positioning accuracy of approximately 0.05 mm [31]. This result indicates that industrial robots do not need to enter high-precision aviation assembly scenarios by simply improving body manufacturing accuracy; a more feasible path is to achieve task-level high precision through heterogeneous sensors and multi-layer compensation.

Around specific process constraints, the industrial robot route has also developed a series of more targeted compensation mechanisms. Lin et al. proposed a hole position correction method based on laser line scanning, enabling the automatic drilling and riveting system to quickly correct reference hole positions before machining [41]. Hu et al. proposed an online laser ranging and offline curvature model-based normal accuracy compensation method for large-curvature variable-curvature panels, controlling the drilling and riveting normal error within 0.4° without adding additional measurement sensors [42]. These studies collectively indicate that industrial robot errors should not be regarded as a single spatial pose deviation, but decomposed into hole position errors, normal errors, contact errors, stiffness deformation, and process load coupling errors, which are governed by multi-layer compensation.

Overall, the most prominent advantages of the industrial robot route are large workspace, strong versatility, high system maturity, and easy integration with process equipment such as drilling, riveting, and inspection [28,29,31]. Its main disadvantages are insufficient body stiffness and absolute accuracy, and strong dependence on external metrology and high-quality calibration [30,32]. Therefore, in the docking assembly of large-scale components, industrial robots are usually most suitable as the core equipment of the execution layer, and form a composite system with high-stiffness support mechanisms, external measurement systems, and special end-effectors, rather than independently undertaking all positioning tasks [15,32].

When comparing NC positioners, PKMs, and industrial robots within the same framework, it can be found that they are not merely three parallel types of equipment, but three distinct precision generation architectures. NC positioners mainly rely on key support points and datum reconstruction to generate precision [20,22]; PKMs tend to front-load precision to the levels of mechanism topology, closed-loop stiffness, and force path [23,24]; industrial robots achieve task-level precision primarily through end-effectors, external measurement, and multi-layer compensation chains [29,30,31,32]. Therefore, their difference does not lie in whether to pursue high precision, but in which level of the system to allocate the precision budget.

From this perspective, regarding the stiffness-precision trade-off between PKMs and industrial robots, there is no strict conclusive conflict between the two; instead, the expressive tension is mostly caused by different research perspectives. The PKM literature emphasizes that high stiffness is a prerequisite for maintaining high precision [23,24,27]; while the industrial robot literature proves that even with insufficient inherent body stiffness, sufficient process precision can still be achieved in specific tasks by configuring high-performance end-effectors, external measurement, and real-time compensation [28,29,30,31]. The former highlights inherent structural capability, while the latter emphasizes system compensation capability—they are not addressing entirely the same issue.

What truly deserves attention is that existing research still lacks a unified comparison framework. Different technical routes typically report results under different tasks, different loads, and different evaluation indicators, making it difficult to directly compare the trade-off relationships between stiffness, precision, measurement dependence, and system cost [1,2,3]. Therefore, to truly promote the development of pose adjustment and positioning technology for large-scale components in future research, the key is no longer merely continuing to improve the performance of a single mechanism, but establishing a unified evaluation system oriented to actual assembly tasks, and systematically comparing the respective precision shares that structural assurance, measurement closed loops, and control compensation should undertake.

### 3.4. Other Novel Mechanisms

In addition to NC positioners, PKMs, and industrial robots, a number of novel mechanisms for special constraints have been developed for the assembly of large-scale aerospace components. The emergence of these mechanisms often corresponds to specific engineering problems that are difficult to solve with traditional equipment, such as insufficient accessibility inside wing boxes, narrow internal fuselage spaces, excessive component compliance, and the shift of production organization from fixed stations to mobile and modularization [17,43]. Therefore, the value of such mechanisms lies not in comprehensively replacing mainstream equipment, but in expanding the boundaries of assembly automation.

Snake arm robots are one of the most representative mechanisms for low-accessibility scenarios. Developed by Anscombe et al. in cooperation with Airbus, this type of system addresses the problem of difficult manual access to internal areas after wing box closure by using highly redundant flexible robotic arms to deliver tools to narrow, bent areas that are inaccessible to traditional robots [33]. The core innovation is not to improve external operation efficiency, but to reconstruct the accessible space of the assembly system. With the gradual development of wing structures towards thinning and composite materials, manual internal operations are not only inefficient, but also accompanied by health and safety risks and structural damage risks [33]. Through a long and compliant continuous arm body, Snake arm provides a new automated channel for tasks such as drilling, fastening, and inspection inside wing boxes, engineeringly reducing the dependence on manual operations in constrained spaces [33].

Mobile heavy-duty mechanisms correspond to another type of demand: scenarios where large-scale fixed devices are not suitable for deployment inside large fuselages or cabin sections. The semi-passive heavy-duty mobile robotic system proposed by Menon and Asada aims to realize automated assembly operations inside the aircraft body [34]. The innovation lies in combining a mobile chassis with high load-bearing capacity and a local operation device, enabling the equipment to move with tasks without deploying full-size fixed tooling throughout the entire operation space [34]. Although such systems bring new challenges in mobile platform positioning, stability, and environmental adaptability, their design ideas provide obvious inspiration for the equipment form of future production-line-free and flexible factory-oriented equipment.

The Autonomous Industrial Mobile Manipulator (AIMM) scheme proposed by Neitmann et al. further integrates mobility with active shape compensation capabilities [35]. Aiming at the assembly scenario of highly compliant flap modules, this study proposes using mobile robots to carry lightweight end fixtures for assembly instead of continuing to rely on bulky rigid fixtures [35]. The core innovation is to integrate a Stewart platform inside the end fixture, enabling the end system to compensate for manufacturing deviations and component shape deviations caused by gravity under closed-loop control [35]. A 1:1 scale experimental verification was completed in the paper [35]. This indicates that an important direction in the future assembly of large-scale flexible components may not be to continue enlarging fixed fixtures, but to transfer active shape compensation capabilities to mobile end-effectors, thereby balancing flexibility and accuracy simultaneously.

The automated reconfigurable robotic fuselage wall panel final assembly system proposed by Iç et al. reflects another path of novel mechanism development: from single-machine innovation to systematic modular reconfiguration [36]. This solution integrates stringer positioning robots, 3D projection, reconfigurable fixtures, mobile feeding friction stir welding robots, robotic measurement systems, and modular lifting equipment into a set of reconfigurable final assembly systems [36]. The innovation lies not in the individual performance of a single device, but in improving the system’s ability to adapt to wall panel model changes, order quantity fluctuations, and delivery rhythm changes through the reorganization of modular assembly resources [36]. This is highly consistent with the flexible unitized production concept emphasized by Assembly 4.0 and production-line-free assembly.

In addition, research on automatic riveting and assembly auxiliary systems for large wall panels also shows that novel mechanisms often appear in process-specialized forms. Webb et al.’s work on flexible automatic riveting of fuselage skin wall panels demonstrates the integration path between flexible automated systems and the beat requirements of large wall panel assembly [44]. Müller et al. further emphasized that reducing dependence on fixed assembly resources through reconfigurable fixtures and auxiliary systems is an important direction for the evolution of large-scale component assembly systems towards flexibility [17]. These studies collectively indicate that “other novel mechanisms” are not marginal supplements, but key technical reserves that provide constrained space operation capabilities, mobile deployment capabilities, and system reconfiguration capabilities for future large-scale component assembly beyond traditional heavy-duty tooling and general robots.

Comprehensively, the four types of pose adjustment and positioning mechanisms do not replace each other, but form a hierarchical collaborative system in the docking assembly of large-scale components [1,2,15]. NC positioners are more suitable for undertaking high-load datum support and slowly changing reconfiguration tasks; PKMs are suitable for undertaking high-stiffness multi-DOF docking and process collaboration tasks; industrial robots are suitable for undertaking large-range operations and integrated drilling and riveting machining tasks; and other novel mechanisms are used to expand the automation boundaries in constrained spaces, mobile assembly, and new factory organization modes [21,25,29,35]. Therefore, the mainstream form of future automated assembly of large-scale components is likely not to be dominated by a single mechanism, but a composite assembly system based on a unified coordinate datum, external metrology closed-loop, and heterogeneous equipment collaboration [2,43].

## 4. Digital Measurement Technologies for Docking Assembly of Large-Scale Components

Digital measurement for the docking assembly of large-scale aerospace components is essentially not just acquiring geometric coordinates, but continuously providing traceable spatial information for pose adjustment, registration, gap control, and quality judgment. Compared with conventional precision manufacturing, this scenario is characterized by large-scale, low-stiffness, strong-occlusion, multi-station, multi-sensor, and on-site environmental fluctuations. Therefore, the value of a measurement system depends not only on instantaneous accuracy, but also on measurement field construction capability, update frequency, adaptability to occlusion and environmental disturbances, and whether uncertainty can be analyzed and transmitted to assembly quality evaluation [2,4,5]. Over the past decade, the technical main line in this field has gradually shifted from single high-precision instrument measurement to a measurement infrastructure composed of vision, laser, large-scale coordinate measurement equipment, and multi-sensor fusion [4,45,46].

From a technical form perspective, digital measurement in the docking assembly of large-scale components can be roughly divided into five categories:

1. Vision measurement: It is characterized by low-cost, multi-feature, direct perception of curved surfaces and pose information, suitable for constructing large-range vision measurement fields.
2. Large-scale measurement equipment (e.g., laser trackers, iGPS, and liDAR): It emphasize coordinate traceability and large-scale spatial coverage, serving as high-precision backbone equipment in assembly workshops [4,47,48].
3. Measurement field construction technology: It solves the consistency problem between multi-station, multi-equipment, and multi-coordinate systems, determining whether the measurement system can be upgraded from local measurement to factory-level spatial infrastructure [49,50].
4. Measurement uncertainty analysis: It further converts “what is measured” into “how credible the measurement is”, serving as the basis for assembly tolerance closed-loop and quality decision-making [8,51].
5. Other technologies (portable photogrammetry, laser scanning, Light Detection and Ranging (LiDAR), structured light, etc.): They rapidly supplement complex curved surface, local detail, and online point cloud positioning capabilities [52,53,54].

Table 2 provides a comprehensive comparison of representative digital measurement technologies covered in this chapter. It should be noted that the cost, update frequency, uncertainty analyzability, and technological maturity in the table are relative judgments, mainly summarized based on the system structure, deployment mode, verification level, and industrial application reported in the literature; the measurement range and accuracy prioritize representative results that can be directly cited in the literature.

It should be noted that the three evaluations (anti-occlusion capability, cost, and update frequency) in Table 2 adopt relative grading standards oriented to the docking assembly scenarios of large-scale components, rather than absolute indicators divorced from specific task conditions.

Among them, anti-occlusion capability is mainly classified according to the measurement system’s ability to maintain effective observation under the conditions of large component dimensions, obvious local shielding, and significant interference from station equipment: those that can still stably acquire key measurement information under multi-view layout, network coverage, or multi-station stitching are classified as “High”; those that can mitigate occlusion impacts by adjusting station positions or measurement strategies under general working conditions are classified as “Medium”; and those highly dependent on a single line of sight, single-station layout, or direct target viewing are classified as “Low”.

Cost is relatively evaluated at the system level, including not only instrument procurement costs but also station deployment, calibration, supporting software, on-site implementation, and maintenance costs: those with high initial investment, complex system deployment, and high maintenance thresholds are classified as “High”; those with generally controllable hardware and implementation costs are classified as “Medium”; and those with relatively low equipment and application costs are classified as “Low”.

Update frequency is mainly graded according to the speed at which the system outputs effective measurement results usable for assembly decisions, rather than merely the sensor sampling frequency itself: those capable of supporting online continuous updates or high-frequency pose feedback are classified as “High”; those suitable for quasi-real-time measurement, phased updates, or result refreshing between multiple process steps are classified as “Medium”; and those mainly applicable to offline inspection, low-frequency updates, or tasks with long measurement cycles are classified as “Low”.

Therefore, the purpose of the relevant grades in the table is to illustrate the typical capability boundaries of different digital measurement technologies in engineering applications, facilitating horizontal comparison of their applicable roles in large-scale component assembly, rather than being interpreted as fixed conclusions on the performance of any specific equipment.

### 4.1. Vision Measurement

Vision measurement is one of the most expandable technologies in the assembly of large-scale components, as it can simultaneously provide feature recognition, pose estimation, surface reconstruction, and multi-point parallel observation at a lower hardware cost. Compared with traditional single-point coordinate measurement, the real advantage of vision methods is not only “seeing more points”, but also integrating curved surfaces, boundaries, hole positions, normals, and overall component poses into the same perception framework [56,59]. This makes vision measurement particularly suitable for serving the docking assembly of large curved components such as fuselage sections, cabin sections, and airfoils.

Early industrial photogrammetry has proven its usability in aircraft pose detection and assembly alignment. Jung and Lee used industrial photogrammetry to conduct alignment inspection on O-2A aircraft, indicating that geometric evaluation of aircraft shape and alignment status can still be supported based on on-site marker points and image reconstruction in the absence of complete design datum data [72]. The significance of such work is to advance vision measurement from an offline detection tool to a geometric reference source in the assembly site. Subsequently, Clarke et al. proposed a verification method for photogrammetry systems, extending the idea of International Organization for Standardization (ISO) 10360-2 to large-volume photogrammetry equipment [70]. The core innovation is not only to provide a calibration process, but also to emphasize that photogrammetry systems must accept parametric error modeling and performance verification like coordinate measurement equipment, thus establishing a traceability basis for their entry into high-value manufacturing scenarios [70].

Vision measurement truly enters the stage of automated assembly of large-scale components after the maturity of large-field-of-view calibration, multi-camera collaboration, and algorithm systems for pose measurement. Zhang et al. proposed a high-precision calibration method based on virtual 3D targets and laser trackers for large-field-of-view binocular vision systems [55]. The core innovation of this method is no longer relying on large-scale physical calibration plates that are difficult to manufacture, but using laser trackers to construct a virtual 3D calibration field covering the entire workspace, while introducing high-order distortion models and Levenberg–Marquardt optimization [55]. In its verification system, the binocular vision field of view reaches approximately 4 × 3 × 2 m^3^ [55]. The engineering value of this work is clear: it solves the key bottleneck of “larger scale, more difficult calibration” for large-field-of-view vision systems, enabling vision systems to expand from small-range experimental devices to large-scale component assembly areas.

For the assembly of large fuselage and cabin sections, multi-camera measurement systems further reflect the systematic advantages of vision methods. Valencia et al. proposed a multi-camera metrology system for shape and position correction of large fuselage components [58]. Different from monocular or binocular local measurement, its core idea is to splice local surface information into overall component state estimation through multi-view observation, thereby serving both shape correction and pose adjustment [58]. This shift from “point measurement” to “component state estimation” is crucial, as assembly failure of large-scale components is often not caused by single-point deviations, but jointly determined by overall pose, local deformation, and contact boundaries.

In scenarios with higher real-time requirements on the assembly site, depth cameras and binocular stereo vision have become important branches of vision measurement. Yang et al. proposed a large aircraft pose estimation system based on depth cameras, achieving complete machine pose solution through multi-directional scanning, point cloud registration, and feature description, with an angular measurement accuracy of approximately 0.05° in experiments [57]. This result indicates that depth vision already has the ability to enter engineering applications in scenarios that are more sensitive to angle and pose updates and have slightly lower absolute length requirements than laser trackers. The binocular vision Six-Dimensional (6D) pose measurement research of cabin sections by Wei et al., and the large-scale component docking recognition and positioning method based on vision measurement fields by Kan et al., further indicate that multi-camera vision fields can support 6D pose recognition and online docking of large curved components at a lower hardware cost [56,73].

Another key progress of vision measurement in engineering applications is the shift from pure geometric reconstruction to “state estimation directly coupled with assembly control”. Liu et al. combined distance sensors with Charge-Coupled Device (CCD) cameras for pose alignment of aircraft structural components [59]. The core innovation is to use different types of vision and distance information to undertake boundary perception and geometric constraint recognition separately, thereby improving the stability of pose solution in docking scenarios [59]. From an engineering effect perspective, such methods are more suitable for large curved components than purely relying on a small number of discrete feature points, as they are more robust to curvature changes and insufficient local features.

Overall, the core advantages of vision measurement are rich observation information, flexible deployment, high update frequency, and easier coverage of complex curved surfaces and local features [55,56]. Its shortcomings are that the results are highly dependent on calibration quality, line-of-sight occlusion, lighting conditions, and image processing algorithms, resulting in a more complex uncertainty composition than laser trackers [52,70]. Therefore, in the assembly of large-scale components, vision measurement is usually more suitable for combined use with laser trackers, iGPS, or laser scanning, with the vision system undertaking rapid pose recognition and surface perception, and high-precision coordinate equipment undertaking global datum constraints [50,59].

Synthesizing the relevant research in Section 3.1 reveals that the true progress of vision measurement technology in the docking assembly of large-scale components lies not merely in the increase in the number of cameras or reconstructed points, but in the gradual shift of measurement objects from discrete geometric points to the overall assembly state. Early industrial photogrammetry has proven that vision-based methods can support aircraft shape and assembly alignment evaluation [70,72], while subsequent research on large-field-of-view calibration, multi-camera collaboration, and depth vision has further advanced vision systems from offline inspection tools to perceptual infrastructure that can directly serve pose recognition, shape correction, and online docking control [55,56,57,58]. In other words, the core value of the vision measurement route does not lie in replacing traditional coordinate measurement equipment to provide single-point high-precision results, but in delivering more comprehensive component state information at a relatively low cost.

The further comparison of different studies shows that the advantages and limitations of vision measurement exhibit strong homology. Precisely because it can simultaneously acquire rich information such as boundaries, curved surfaces, hole positions, normals, and overall poses [56,59], it is particularly suitable for docking scenarios of large curved components and complex interfaces; however, precisely because it is highly dependent on calibration models, field-of-view layout, feature extraction, and lighting conditions, its error sources are often more scattered than those of equipment like laser trackers and harder to directly compress into a single precision index [52,70]. Therefore, an implicit consensus has actually formed in existing literature: vision measurement is more suitable as a “state perception layer” rather than a standalone “global datum layer”, and its engineering competitiveness mainly stems from information richness and update frequency, not absolute coordinate precision itself.

Nevertheless, there are obvious gaps in this direction. Most studies focus on verifying pose recognition accuracy, field-of-view calibration accuracy, or the feasibility of online measurement under specific scenarios [55,57,73], while discussions on how vision results are stably transmitted to assembly quality criteria—especially how to establish repeatable correlations with gap control, contact states, and subsequent process consistency—remain insufficient [58,59]. This indicates that vision measurement technology has well addressed the question of “how large-scale, complex surfaces can be quickly perceived”, but existing research has not yet formed a fully mature unified framework for “how perceptual results can reliably support high-value assembly decisions”.

### 4.2. Large-Scale Measurement Equipment

Large-scale measurement equipment is the backbone metrology infrastructure in large-scale component assembly workshops, with the common feature of providing traceable coordinate information on a scale of several meters to tens of meters. Compared with vision systems, such equipment usually has more mature error models, clearer calibration specifications, and higher coordinate credibility, thus undertaking tasks such as assembly datum establishment, key point measurement, equipment calibration, and final geometric acceptance for a long time [4,48]. Among them, laser trackers, iGPS, and liDAR are the three most representative technical routes.

Laser trackers are one of the most widely used equipment in current large-scale aerospace assembly. The review by Muralikrishnan et al. points out that laser trackers have become the core equipment for large-volume dimensional metrology and are widely used in aviation assembly, tooling calibration, and machining process verification [4]. Their engineering advantages are not only high measurement accuracy, but also portability, re-deployability, and the ability to cover large-scale spaces through multi-station networks [4]. For their performance verification, Clarke et al. proposed extending the idea of ISO 10360-2 to large-volume measurement systems [62], while Muralikrishnan et al. and Nasr et al. studied the sensitivity and optimization of laser tracker performance evaluation to geometric misalignment based on American Society of Mechanical Engineers (ASME) B89.4.19 [61,63]. The core value of these studies is to provide a standardized path for laser trackers to transform from “high-precision equipment” to “verifiable, comparable, and traceable industrial metrology equipment” [61,63].

In large-scale assembly sites, the real engineering role of laser trackers is not to measure a few points at a single station, but to serve as datum equipment for large-scale coordinate networks. In the iGPS capability study by Hughes et al., a reference system was constructed using a laser tracker multi-lateration network, achieving approximately 10 µm-level expanded uncertainty of reference coordinates in a volume of approximately 10 m × 10 m × 2 m, k = 2 [60]. This result is often regarded as a representative level of large-scale high-precision reference networks, and also indicates that under ideal configuration and strict modeling, laser tracker networks can provide high-confidence datums for the calibration and verification of other equipment [60]. At the same time, Mur09 and Nas12 also clearly pointed out that laser tracker performance is highly sensitive to internal mechanical and optical misalignment, angle encoder quality, thermal effects, and atmospheric compensation [61,63]. Therefore, the high precision of laser trackers is not a natural attribute of the equipment, but a systematic result based on calibration, verification, and environmental control.

iGPS represents another large-scale measurement idea. Maisano et al. pointed out that iGPS realizes large-scale spatial positioning through multi-transmitter networks, and its relatively portable, reconfigurable, and easy-to-install features make it particularly suitable for large-scale manufacturing environments [47]. Compared with single laser trackers, the core innovation of iGPS is not higher single-point measurement accuracy, but its network structure is naturally suitable for covering large volumes and serving dynamic tracking and multi-object positioning [47,64]. The capability study by Hughes et al. shows that in a test volume of approximately 10 m × 10 m × 2 m, iGPS can measure lengths up to 12 m, with a length uncertainty of approximately 170 µm and a coordinate uncertainty of approximately 120 µm × 120 µm × 190 µm, k = 2 [60]. Although this level is lower than that of laser tracker reference networks, it is sufficient to meet the online positioning and process feedback requirements in many aviation assemblies.

The engineering significance of iGPS lies in its greater suitability as a “continuous spatial perception system” for production processes rather than a single high-precision acceptance equipment. The deployment test of Wang et al. in large-scale production facilities indicates that iGPS has sub-millimeter-level accuracy potential and good repeatability, but large workshop scales and environmental factors will significantly increase measurement uncertainty [64]. Mosqueira et al. used iGPS as a robot feedback source in fuselage alignment and compared it with liDAR results, indicating that iGPS can be used for real-time control closed-loop in the docking of large-scale components [66]. Heiden et al. further verified in simulated measurement-assisted assembly experiments that iGPS has practical applicability for assembly processes with production tolerances of 1 mm [68]. This means that iGPS does not aim to replace laser trackers, but is more suitable as a global dynamic positioning platform in the process.

LiDAR and automated scanning measurement equipment reflect the trend of large-scale metrology towards automation and densification. Muelaner and Maropoulos listed liDAR as an important technical route parallel to laser trackers in the review of large-volume measurement technologies, emphasizing its suitability for large-scale, complex surfaces, and non-contact measurement scenarios [48]. Taking Saab’s automated liDAR system as an example, Linqvist et al. proposed using digital twins and offline simulation to plan large-scale measurement processes, thereby verifying line-of-sight accessibility and rationality of measurement sequences at the scheme design stage [46]. The core innovation is not the adoption of new equipment, but the digitization of measurement system planning itself, thereby reducing on-site trial and error and improving the efficiency of automated measurement deployment [46]. Although such equipment still often needs to rely on external datum networks for absolute coordinate traceability, they have obvious advantages in complex surface shaping, automated coverage, and offline programming.

Overall, the division of labor of large-scale measurement equipment is relatively clear: laser trackers are suitable for high-precision datum measurement, calibration, and final verification; iGPS is more suitable for large-scale online positioning and dynamic feedback; liDAR is more suitable for automated dense measurement and complex surface acquisition [4,46,60]. The mainstream future trend is not the dominance of a single equipment, but the collaborative work of different large-scale equipment under a unified coordinate datum, undertaking different roles such as datum establishment, online tracking, and surface perception [46,50].

From cross-study comparisons, the development of large-scale measurement equipment has not evolved around a single type of instrument in isolation, but has gradually formed a measurement framework system with clear division of labor. Laser trackers emphasize high precision, traceability, and standardized verification capabilities, making them more suitable as core equipment for datum establishment, equipment calibration, and final geometric acceptance [4,47,61]; iGPS emphasizes network coverage, dynamic tracking, and online feedback capabilities, thus being more suitable as a large-space continuous positioning platform [47,64,66,68]; liDAR and automated scanning systems are better suited for complex curved surfaces and large-scale automated measurement tasks [46,48]. Therefore, what has truly formed in this field is not an equipment substitution relationship, but a gradually clarified functional stratification centered on three types of tasks: “datum establishment, dynamic tracking, and surface acquisition”.

Further analysis shows that there is no simple precision superiority–inferiority relationship between the laser tracker and iGPS literature, but rather two distinct engineering philosophies. The laser tracker route emphasizes ensuring coordinate credibility through rigorous calibration, performance verification, and environmental control [61,62,63], with the logic of first establishing a high-credibility datum and then expanding the measurement network outward. The iGPS route, on the other hand, places greater emphasis on maintaining continuous spatial perception capabilities in large-scale manufacturing environments [47,60,66], and its core competitiveness lies not in the highest single-point precision, but in networking, real-time performance, and multi-target coverage capabilities. In other words, the former is closer to a “high-precision datum device”, while the latter is more like a “workshop-level online spatial infrastructure”—they serve not entirely the same measurement propositions.

Nevertheless, existing research collectively reveals a key issue: the performance of large-scale measurement equipment is often highly dependent on system configuration rather than the nominal indicators of the equipment itself. Whether it is the sensitivity of laser trackers to internal misalignment, thermal effects, and atmospheric compensation [61,63], or the dependence of iGPS on network layout and environmental factors [47,68], it indicates that measurement credibility in large-scale component assembly is essentially a system-level result, not an inherent attribute of a single machine. Therefore, the true research direction suggested by the relevant literature in Section 3.2 is not to continue pursuing the performance improvement of individual equipment in isolation, but to establish a unified evaluation perspective oriented to assembly tasks, comprehensively comparing equipment precision, spatial coverage, online capability, environmental adaptability, and network topology within the same framework [4,46,60].

### 4.3. Measurement Field Construction

In the assembly of large-scale components, the accuracy of a single equipment is not equivalent to the accuracy of the entire measurement system. What really determines assembly usability is often whether a stable and consistent measurement field can be formed between multi-station, multi-equipment, and multi-coordinate systems. The problem solved by measurement field construction technology is not “whether a certain equipment measures accurately”, but “whether the coordinate relationship in the entire workshop is stable, traceable, and expandable” [49,50]. This is why the same laser tracker may lead to significant differences in assembly results under different datum point layouts and network configurations.

Enhanced Reference System (ERS) is a typical infrastructure for measurement field construction in large-scale assembly. Jin et al. systematically analyzed the influence of ERS point configuration on transformation matrix errors, established an explicit error estimation model, and accordingly provided guiding principles for ERS layout, volume size, quantity, and selection of assembly coordinate system position and orientation [74]. The core innovation of this work is to transform the datum point layout problem, which was mainly dependent on experience in the past, into a quantifiable and optimized problem. Its engineering value is direct: in the assembly of large-scale components, improper datum point layout will significantly amplify multi-station fusion and coordinate transformation errors, while optimized ERS can significantly improve the overall stability of the measurement field [74].

For multi-station laser tracker networks, Den et al. proposed a Hybrid Reference System (HRS) to solve the problem of excessive transformation uncertainty when standard datum points cannot effectively envelope key assembly areas [75]. The innovation lies in introducing a small number of temporary extension points without nominal coordinates to enhance the rigidity of the network in weak areas, and deriving the analytical solution of transformation parameter uncertainty based on the extended Gauss–Markov model [75]. In the on-site measurement application of the tail beam, only by adding three temporary extension points, the average transformation uncertainty was reduced by 26% and dropped below 0.05 mm [75]. This result indicates that the accuracy of the measurement field does not entirely depend on the upgrade of the instrument body, and the network geometric design itself is a cost-effective means to improve accuracy.

Gai et al. further proposed Bundle Adjustment for Multi-Station (BAMS) to replace the traditional single-station best-fit construction method [49]. This method incorporates all stations and ERS into a unified measurement coordinate system, establishes the overall Extended Measurement Coordinate System (EMCS) through bundle adjustment and then realizes registration to the assembly coordinate system through stable ERS points [49]. The core innovation is shifting from the local idea of “station-by-station splicing” to the systematic idea of “global joint estimation”, thereby reducing the sensitivity to single-station and local datum point errors [49]. Both simulations and experiments in the paper show that BAMS can more effectively constrain system uncertainty than BFSS [49]. This idea is particularly important for long-scale components such as large wings, fuselage sections, and rocket cabin sections, because local optimality is often not equal to global optimality.

With the development of Assembly 4.0 and production-line-free assembly, measurement field construction has risen from a station-level problem to a factory-level problem. Huber et al. proposed a Global Reference System (GRS) for integrating “global” large-scale sensors and “local” high-precision sensors within the factory scope [50]. The method unifies the coordinate systems of each measurement device through homogeneous transformation, then fuses pose estimation using Kalman filtering and gives the covariance of the fusion result in a Guide to the Expression of Uncertainty in Measurement (GUM)-compatible manner [50]. The core innovation of this work is to organize originally scattered local metrology equipment into a spatial infrastructure that can continuously provide unified position data, thereby serving automatically reconfigurable production lines and mobile assembly units [50]. This means that measurement field construction is no longer just a matter of “arranging a few datum points”, but a spatial information architecture design for the entire assembly system.

Measurement field construction is also closely related to measurement process planning and line-of-sight analysis. Lindqvist’s research shows that offline simulation based on 3D models and digital twins can verify laser paths, line-of-sight accessibility, and rationality of measurement sequences before system deployment, thereby reducing the difficulty of actual measurement system debugging [46]. This is crucial for the assembly of large-scale components, because occlusion, inclination restrictions, and equipment repositioning are often the main bottlenecks for on-site measurement efficiency and stability. In other words, the quality of a measurement field depends not only on the geometric error model, but also on whether the measurement process is systematically planned.

Overall, the core task of measurement field construction technology is to transform several isolated high-precision pieces of equipment into a stable, unified, and expandable spatial measurement infrastructure [49,50,74]. Its engineering effects are mainly reflected in three aspects:

1. Reducing multi-station and multi-equipment fusion errors.
2. Improving effective accuracy in key assembly spaces.
3. Supporting mobile, flexible, and factory-level automated assembly [46,50,75].

This level of capability often determines the actual value of a large-scale component measurement system more than the nominal accuracy of a single instrument.

### 4.4. Measurement Uncertainty Analysis

Measurement uncertainty analysis is the most underappreciated yet critical aspect for assembly quality in digital measurement of large-scale components. The reason is that assembly of large-scale components does not simply compare the gap between measured and nominal values; instead, it determines whether to continue pose adjustment, allow docking or require fitting or compensation based on measured position, pose, gap, and surface profile information. If these decisions rely solely on point estimates while ignoring uncertainty, the system may misjudge acceptable deviations as out-of-tolerance or misclassify high-risk boundary states as qualified [8,76]. Therefore, the true value of uncertainty analysis is not to add an auxiliary note to measurement results, but to convert measurement results into credible information usable for assembly decision-making.

Chen et al. proposed a multi-dimensional U-P&O representation and analytical algorithm for position and pose uncertainty in measurement-assisted alignment, systematically discussing its influencing factors and role in assembly quality evaluation [8]. The key contribution of this work is to unify influencing factors traditionally scattered in coordinate errors, angular errors, and registration errors into a position and pose uncertainty framework, enabling assembly quality assessment to directly target the docking task itself rather than only local measurement points [8]. Du’s research further shows that evaluation of connection quality for large-scale components must directly consider position and pose measurement uncertainty; otherwise, assembly decisions can hardly reflect real geometric risks [76]. This indicates that in large-scale assembly, uncertainty is not the end of the measurement phase, but the starting point of the quality control phase.

Table 3 summarizes the primary uncertainty sources and their propagation paths in digital measurement of large-scale components.

From the perspective of error sources, the first category is sensor intrinsic errors. For laser trackers, Muralikrishnan demonstrated that small offsets, tilts, and eccentricities in mechanical and optical components introduce systematic errors in spherical coordinate measurements, ultimately affecting reference lengths and spatial coordinates [63]. Huo et al. proposed the Virtual Laser Tracker framework, whose core innovation is to systematically organize laser tracker error sources into a virtual measurement model that can be modeled, combined and propagated [77]. The significance of this framework lies in transforming the empirical perception that “equipment has errors” into an engineering approach where “errors can be modeled item-by-item and included in the uncertainty budget” [77]. For scenarios requiring multi-station and multi-equipment collaboration such as aviation assembly, analysis at the network and fusion layers can only be grounded if error mechanisms are clearly defined at the sensor level.

The second category is calibration residuals and incomplete modeling, which are not covered by nominal equipment accuracy. Large-scale vision systems are a typical example. Zhang et al. showed that traditional planar calibration can hardly ensure full-field accuracy for large-field-of-view vision systems and hence the introduction of a calibration method based on virtual 3D targets and high-order distortion models [55]. This implies that when the measurement volume expands to the meter scale, residual distortion, coupling of camera extrinsic parameters, and calibration geometric degradation amplify rapidly. Similarly, in multi-laser-tracker networks, treating stations and reference points as fixed parameters without estimation in a unified model directly introduces calibration errors into the assembly coordinate system [49].

The third category is environmental factors, which are often the most difficult uncertainty sources to completely eliminate in large-scale on-site measurement. Nasr pointed out that the uncertainty of laser trackers is affected not only by internal alignment and angular scale quality, but also directly by atmospheric compensation, thermal expansion of instruments and mounts, and thermal deformation of measured workpieces [61]. Muñoz et al. specifically analyzed the effects of the initial thermal stabilization phase and air turbulence of laser trackers on measurement results, showing that equipment warm-up stabilization and on-site airflow variations significantly impact result stability [79]. Muelaner et al. further noted in a study on benchmark measurement of large-scale aero-engines that after process optimization, the dominant uncertainty source is no longer the instrument itself, but workpiece thermal expansion, that is, without a temperature-controlled environment, simply improving instrument accuracy yields no proportional benefits [51]. This conclusion is universally relevant to large-scale aerospace assembly, as the on-site environment often determines final credibility more than nominal instrument specifications.

The fourth category is network geometry and coordinate transformation errors. Assembly of large-scale components often requires joint measurement by multi-station equipment, and coordinate transformation uncertainty varies significantly with reference point configuration, station layout, and critical assembly spatial positions [74,75]. Deng’s HRS research showed that adding only three temporary extension points reduced the average coordinate transformation uncertainty by 26% to below 0.05 mm [75]. This result indicates that uncertainty is not always resolved by instrument upgrades, but often depends on whether the reference network reasonably envelopes critical spaces. Gai’s BAMS research also showed that multi-station bundle adjustment better constrains system uncertainty than single-station best fitting [49]. Therefore, for large-scale assembly systems, network geometry itself is an object of uncertainty control, not a fixed background condition.

The fifth category is uncertainty introduced by multi-sensor fusion and data processing. Chen analyzed the measurement principle and uncertainty of a large-volume measurement network combining iGPS and portable scanners, showing that when multiple sensors collaborate in the same task, errors are no longer simply superimposed but recoupled through reference frame transformation, registration algorithms, and data fusion processes [80]. Huber used Kalman filtering to fuse local and global sensors in the GRS framework and provided GUM-compliant uncertainty representation via covariance matrices [50]. Yang also pointed out that in wing assembly gap measurement, the point cloud registration process itself introduces considerable errors, which are significantly affected by software parameter settings [69]. Kaufmann’s research on uncertainty propagation in virtual assembly further showed that constrained nonlinear registration algorithms propagate greater uncertainty than unconstrained Gaussian best fit, indicating that the more complex modern assembly algorithms become, the more unavoidable uncertainty propagation is [81].

The sixth category is measurement strategy and operator factors. Unlike small-scale laboratory metrology, large-scale assembly measurement often heavily relies on on-site layout, sampling paths, measurement point selection, and operator experience. Puerto et al. evaluated the performance of portable photogrammetry in 3 m to 64 m scenarios, specifically treating equipment spatial layout and camera relative poses as key process variables, and studied performance differences between skilled and unskilled users through Monte Carlo analysis and experimental verification [52]. Yang’s task-specific laser tracker uncertainty research also noted that measurement network fusion, measurement strategies, process factors, and operators are non-negligible error sources [9]. The common significance of such studies is expanding uncertainty analysis from “instrument error analysis” to “measurement process error analysis”.

From the perspective of propagation paths, measurement uncertainty of large-scale components is typically amplified or redistributed step-by-step along the following chain: uncertainty of raw observations is first converted into 3D coordinate uncertainty via geometric models, then transmitted into assembly coordinate system uncertainty through multi-station registration and reference frame transformation, and further propagated in feature extraction, pose solving, gap calculation, and quality judgment [8,50,75,81]. This means that what ultimately affects assembly decisions is not the individual error of a single sensor, but the composite propagation process of the entire “observation model to decision model”. For tasks such as wing docking, cabin segment docking, and large-scale curved surface fitting, this composite propagation is often more critical than single-point measurement errors [69,76].

In terms of uncertainty quantification frameworks, Guide to the Expression of Uncertainty in Measurement (GUM) and its analytical propagation approach remain foundational in industrial metrology, as they quickly yield results via variance-covariance propagation and are particularly suitable for coordinate transformation, sensor fusion, and nearly linear models [50,75,82]. Deng’s research showed that the HRS uncertainty solution based on analytical covariance propagation is consistent with Monte Carlo results but features simpler solving and lower computational cost [75]. Huber also adopted GUM-compliant covariance representation in GRS, enabling fused pose estimation to directly enter system-level uncertainty management [50]. Thus, GUM still holds clear engineering advantages in network geometry design, online filtering, and factory-level sensor fusion.

However, as measurement models for large-scale component assembly become more complex, especially involving nonlinear registration, constrained optimization, point cloud processing, and task-specific evaluation functions, relying solely on linearized propagation is often insufficient. The Monte Carlo method, represented by JCGM 102, shows stronger adaptability in such problems [9,52,83]. Wang applied Monte Carlo to laser tracker measurement uncertainty assessment, demonstrating its suitability for complex measurement models [83]. Yang compared GUF and Monte Carlo in task-specific uncertainty for laser tracking, showing a ~10% difference in results in case studies, with Monte Carlo better suited for complex models, varying sampling strategies, and scenarios where partial differentiation is inconvenient [9]. Jalid also compared GUM and Monte Carlo in form and position error assessment, further showing that Monte Carlo is often more robust for nonlinear and boundary-type evaluation quantities [84]. Kaufmann’s virtual assembly research further proved that propagation results significantly depend on the selected framework when the algorithm itself involves constraints and nonlinearity [81].

Table 4 summarizes the characteristics of commonly used uncertainty quantification frameworks in digital measurement of large-scale components.

Overall, GUM and Monte Carlo are not mutually exclusive but rather complementary tools for different levels of problems. GUM’s analytical propagation remains the mainstay for network design, online fusion, and real-time control; Monte Carlo better reflects real propagation characteristics for registration with complex constraints, vision reconstruction, and task-specific evaluation [9,75,81]. Future uncertainty analysis for large-scale component assembly measurement will increasingly emphasize three points: first, shifting from instrument-level budgeting to process-level budgeting; second, shifting from static acceptance to online propagation; third, shifting from single-sensor analysis to joint multi-sensor and multi-model analysis [46,50].

### 4.5. Other Measurement Technologies

In addition to vision systems and large-scale coordinate equipment, portable photogrammetry, terrestrial laser scanning, LiDAR, structured light, and local positioning technologies based on point cloud registration are becoming important supplements to the measurement system for large-scale component assembly. A common feature of these technologies is their ability to acquire curved surfaces, edges, and hole features with high data density, making them particularly suitable for complex contours, local tooling identification, gap measurement, and rapid reconstruction of local areas [52,53]. They do not necessarily replace high-precision reference equipment such as laser trackers but play a significant role in enriching perception dimensions and improving on-site efficiency.

The prominent advantages of portable photogrammetry are flexible deployment and low cost. Pueyo et al. noted that this technology has been applied to large-volume scenarios of approximately 3 m to 64 m, but its performance significantly depends on equipment layout and operator experience [52]. The core innovation of this study is not only reporting measurement errors but designing a methodological system combining Monte Carlo and experimental verification, incorporating camera spatial layout and triangular intersection poses as key process variables into the analysis [52]. This elevates portable photogrammetry from an “empirical low-cost solution” to a “process-based metrological solution that can be evaluated and optimized”. For large-scale component assembly, this method is particularly suitable for rapid measurement in areas where high-end fixed equipment cannot be deployed.

Laser scanning and terrestrial laser scanners compensate for the insufficient representation of complex surfaces by discrete point measurement. Pexman and Robson performed multi-station scanning on a wing segment assembly site using a Leica RTC360 (Leica Geosystems AG, Heerbrugg, Switzerland)and achieved point cloud registration using spherical targets compatible with the laser tracker network [53]. The results showed that the point cloud registration accuracy reached the sub-millimeter level, with deviations within 0.2 mm between the center positions of jig holes and the laser tracker reference results [53]. The engineering significance of this work is prominent, as it proves that high-density scanning point clouds can be used not only for visualization but also directly for assembly feature extraction and process object identification. This capability is critical for scenarios involving numerous local tooling and temporary auxiliary parts such as large wings, wall panels, and cabin segments.

LiDAR and point cloud registration technologies further drive large-scale component measurement from “point measurement” to “surface measurement”. Yang pointed out in a study on wing assembly gap measurement that point cloud registration itself introduces non-negligible errors, and software parameter settings significantly affect gap evaluation results [69]. This indicates that when assembly measurement relies on surface-level point clouds and automatic registration, algorithms are no longer just post-processing tools but part of the measurement chain. Jiang et al. proposed a registration framework for non-uniform LiDAR scanning and large pose changes for aircraft assembly, noting that LiDAR’s advantage lies in rapidly acquiring complete surfaces containing deformation information, but non-uniform distribution, large pose changes, and insignificant features significantly increase registration difficulty [54]. This trend shows that the competitive focus of future large-scale component assembly measurement is gradually shifting from equipment hardware to the overall capability of “hardware + registration algorithms”.

Structured light and coded light projection also play roles in automatic cabin segment docking and end coordinate calibration. Hou et al. used coded structured light for cabin segment pose measurement and completed tool control coordinate system calibration through two-stage point cloud registration and AX = XB solving [71]. The core value of such methods is directly coupling local high-density 3D reconstruction with robot calibration and docking control, thereby reducing traditional manual alignment and repeated trial processes [71]. Compared with laser trackers, structured light solutions typically have shorter measurement distances and are more sensitive to ambient light but offer clear advantages in local fine feature acquisition and automated unit integration.

Overall, the strategic value of other measurement technologies lies in expanding digital measurement for large-scale component assembly from “a small number of high-precision points” to a multi-layer system coexisting with “dense surfaces, local features, and global references” [52,53,54]. Future more competitive systems will not rely on a single device but use laser trackers and iGPS to provide a global framework, and vision and scanning systems to supply local high-density information, and organize them into an integrated measurement system usable for assembly decision-making through a unified reference frame and uncertainty propagation framework [46,50,69].

## 5. Trajectory Planning and Control for Docking Assembly of Large-Scale Components

The biggest difference between trajectory planning and control in docking assembly of large-scale aerospace components and general industrial assembly is that the control object is often not a single rigid body, but a coupled system composed of large-scale low-stiffness components, reconfigurable tooling, robots, measurement systems, and contact interfaces. For such systems, generating a collision-free trajectory offline is usually far from sufficient, because gravity deformation, clamping errors, local interference, gap distribution, contact forces, and measurement delays continuously change feasible trajectories and optimal control strategies during actual assembly [1,2,7]. Therefore, the main research line over the past decade has not been to simply improve the solving efficiency of trajectory planning algorithms, but to gradually integrate external measurement, contact perception, digital twins, and flexible body modeling into the control loop, shifting trajectory planning from “geometric path generation” to “process decision-making for quality objectives”.

Based on existing research, planning and control technologies in docking assembly of large-scale components can be roughly divided into five routes. Traditional trajectory planning and control emphasize pose solving, step-by-step alignment, hybrid force-position control, and contact constraint handling, forming the most widely used basic framework in engineering applications [86,87]. Multi-sensor fusion methods improve the observability of invisible interfaces and complex contact processes by jointly estimating states through heterogeneous data such as vision, laser tracking, force sensing, and displacement sensing [88,89]. Digital twin methods embed virtual trial assembly, real-time data mapping, and error prediction into the control loop, turning “trial before assembly” from an empirical behavior into an online model-driven process [90,91,92]. Flexible assembly technology further incorporates component deformation itself into planning objects, representing a key direction for assembly of large-scale composite components and thin-walled parts [10,11,93]. In addition, new technologies such as reinforcement learning, constraint programming, single-step shimming, and machine learning gap prediction are transforming the implementation of planning and control [93,94,95,96].

Table 5 provides a comprehensive comparison of the five types of planning and control technologies covered in this chapter. Note that “deformation modeling capability” in the table refers not only to whether elastic deformation is considered, but also to whether the method explicitly incorporates geometric changes caused by local shape errors, gravity deflection, and assembly contact into the control model. Verification levels are divided into simulation, laboratory, and industrial application.

### 5.1. Traditional Trajectory Planning and Control Technologies

Traditional trajectory planning and control technologies remain the foundation of docking assembly for large-scale components, because regardless of whether vision, digital twins or flexible body models are introduced later, the assembly system must ultimately solve three basic problems: how to generate motion paths satisfying spatial accessibility and collision constraints, how to maintain stability upon contact, and how to guide components to the target assembly state in the presence of local deviations [86,98]. The core evolution of this technical route is not a switch from “planning” to “control”, but a gradual transition from pure position-driven to constraint control with contact perception.

In early research, industrial robots relied on preset trajectories and offline calibration to complete assembly actions, but this method struggled to adapt to the geometric discreteness of aviation components and the compliance of robot bodies. Jonsson et al. noted in aircraft structural assembly research that composite manufacturing fluctuations and industrial robot positioning limitations make pure position control unable to stably complete alignment; thus, introducing force feedback allows the system to advance assembly based on contact states rather than solely target coordinates [100]. The core innovation of this work is converting force sensing originally used for collision protection into assembly guidance information, shifting the robot’s goal from “reaching a position” to “achieving a contact state” [100]. The important engineering significance of this route is reducing reliance on special tooling and absolute calibration, making part-to-part assembly practically feasible.

Stolt et al. further systematized this idea, proposing a robot assembly framework based on constrained motion sequences and sensor-triggered switching [86]. The innovation of this framework is not only adding force control but representing assembly tasks as a series of local motion stages constrained by geometric and contact conditions, with state switching driven by thresholds or sensor events [86]. Compared with single continuous trajectories, this approach is more suitable for large-scale component assembly, as real assembly processes often include distinct stages such as searching, contacting, sliding, pressing, and insertion, each with different feasible control laws.

For specific assembly tasks, hybrid force-position control has become a representative traditional method for pose adjustment of large-scale components. Chu et al. pointed out that, for large-scale component pose adjustment systems composed of multiple NC positioners, treating each support unit with traditional position control generates large internal interaction forces in components due to manufacturing and installation errors [87]. The proposed hybrid force-position control method divides position and force control axes based on screw theory, enabling the system to actively suppress internal force accumulation while maintaining pose adjustment capability [87]. The key innovation of this method is treating “pose adjustment accuracy” and “internal force control” as equally important control objectives, rather than only focusing on the final pose error. Experimental results show that this method significantly reduces interaction forces between positioners, thereby improving the assembly quality of large-scale components [87]. Subsequently, Chu21 applied this concept to ball-and-socket positioning scenarios, with experiments showing that hybrid force-position control stabilizes and speeds up ball head positioning, significantly reduces lateral forces, and requires no additional hardware costs compared with traditional methods [99].

In terms of docking geometry optimization, Wang et al. proposed a wing panel assembly gap control method based on pose alignment [97]. Its core innovation is introducing small pose transformation (SPT) to linearize the originally nonlinear relationship between pose and gaps, then redistributing multi-region gaps using a weighted optimization model with tolerance constraints [97]. The significance of such methods lies not only in finding the “optimal pose” but shifting the traditional pose adjustment centered on local positioning points to a global optimization mind centered on final gap distribution and connection quality. Tests based on simulation and real product data in the paper show that this method effectively coordinates mating surface relationships and controls out-of-tolerance gaps within allowable ranges [97].

For insertion and hole-axis fitting tasks, traditional trajectory planning and control have also developed methods combining passive compliance and servo feed. Jiang et al. proposed an automatic assembly scheme of compliant assembly with 5-DOF spatial mechanism fitting and servo feed for large hole-axis fitting structures such as canards and vertical tails [98]. Its core innovation is transforming the problem that “large-scale hole-axis fitting must rely on manual trial” into a control problem of “automatic guidance after entering the chamfer range” through structural compliance and mechanical analysis of assembly stages [98]. Application tests show that the system achieves efficient and high-quality automatic assembly once the shaft end enters the hole chamfer range, and has been applied in actual aircraft assembly projects [98]. Such results indicate that traditional control routes have not lost value with the emergence of new technologies; on the contrary, they remain the most mature in clarifying assembly mechanisms and contact constraints.

Multi-robot collaboration is also an important extension of the traditional control route. Tingelstad et al. completed high-performance aviation component assembly and spot welding using two real-time controlled industrial robots and a high-precision non-contact measurement system, aligning two components to a deviation level of less than 0.1 mm within manufacturing tolerance requirements [102]. The key significance of this work is proving that traditional industrial robots can enter high-precision large-scale component assembly scenarios when combined with high-precision measurement feedback and coordinated control [102]. Tin14’s closed-loop alignment process for aero-engine component assembly reflects the natural integration of traditional control and measurement-assisted closed loops [113].

Overall, the advantages of traditional trajectory planning and control are clear models, mature engineering implementation, and easy coupling with existing process flows [86,98]. Its limitation is that most are based on rigid or local compliance approximations; when component surface errors, gravity deformation, and multi-source measurement uncertainty dominate assembly results, traditional force-position control alone is often insufficient [10,114]. This is why subsequent studies increasingly introduce multi-sensor fusion, digital twins, and flexible body modeling.

### 5.2. Trajectory Planning and Control Based on Multi-Sensor Fusion

A long-standing challenge in docking assembly of large-scale components is that key contact interfaces are often partially invisible, locally unmeasurable, and feature highly nonlinear state changes. Relying on a single sensor leaves the control system lacking global pose information, local contact information, or unable to stably handle occlusion and measurement delays [88,89]. The core value of multi-sensor fusion technology is organizing the observation advantages of different sensors, shifting trajectory planning from “feedback based on a single coordinate source” to “closed-loop decision-making based on multi-source state estimation”.

Zha et al. proposed a heterogeneous measurement equipment data fusion method for automatic wing-fuselage docking, integrating laser trackers, industrial cameras, and pose adjustment mechanisms into a unified system [88]. Its core innovations are twofold. First, establish wing pose adjustment state equations, laser tracking observation equations, and industrial camera observation equations separately, so that different sensors no longer merely provide parallel data but assume distinct observation roles in a unified system model [88]. Second, combine evolutionary particle filtering on the basis of weighted least-squares fusion to track and estimate wing pose adjustment control points [88]. This allows the control system to utilize both the high-precision global information of laser trackers and the continuous perception of local states by vision systems, making it more suitable for handling visibility changes and multi-stage pose adjustment during docking.

Yu and Du proposed a heuristic alignment method based on multi-source data fusion for cabin components [89]. The key idea of such methods is not simply improving perception accuracy, but narrowing the feasible pose space through multi-source observations under incomplete information and partially invisible interfaces [89]. This is particularly important for large fuselage sections, rocket cabin segments, and barrel components, as the control system must solve not a deterministic geometric inversion problem but a process optimization problem with uncertain boundaries and partial observation conditions.

An important shift in the industrial application of multi-sensor fusion technology is moving from “sensor stacking” to “joint design of estimation and control”. Li’s work, although more focused on force-position control, essentially embodies a unified modeling mind for workspace information, contact force information, and pose control [103]. Similarly, many large-scale component pose adjustment systems have gradually formed a hierarchical architecture: global measurement equipment provides coarse positioning and pose constraints, local vision or displacement sensors provide fine alignment information, and force sensors suppress impacts and constraint overshoot in the final contact stage [88,113]. This hierarchical fusion is effective because state information requirements differ across stages: global position error is prioritized at long distances, while local normal and contact states matter more near contact.

From an engineering perspective, the greatest value of multi-sensor fusion is making invisible and unmeasurable assembly processes “indirectly observable” [88,89]. It does not necessarily improve the accuracy of each sensor individually but reduces blind spots in the control system and improves the robustness of trajectory adjustment and contact transition through complementarity. The main challenges are time synchronization of heterogeneous data, coordinate unification, observation delays, noise characteristic differences, and computational costs of fusion algorithms [50,88]. Therefore, this route is highly suitable for linkage with digital twin and measurement field construction technologies, with unified reference frames and virtual models providing stable support for multi-sensor fusion.

### 5.3. Trajectory Planning and Control Based on Digital Twin Technology

Digital twin technology has developed rapidly in docking assembly of large-scale components fundamentally because the traditional “measure-then-control” model cannot fully handle invisible interfaces, process cumulative errors, and multi-physics coupling effects. Many key states in docking assembly—such as gap distribution of insertion interfaces, local interference trends, surface offsets caused by thermal gravity, and whether subsequent trajectories will cause impacts—cannot be fully observed directly from current measurement data [104,105]. The value of digital twins lies in using cyber-physical mapping, process simulation, and online updates to convert currently unobservable information into predictive states usable for decision-making.

Meng et al. proposed a digital twin-driven control method for automatic assembly of large-scale spacecraft components, describing physical attributes of assembly elements via 3D virtual models, constructing behavior models through robot kinematics, and realizing cyber-physical interaction through information models [90]. Its core innovation is embedding PRM collision-free path planning into the digital twin control architecture, enabling trajectory planning to no longer stay in offline planning software but directly drive physical robots to perform assembly tasks through virtual models [90]. Experimental results show that this method safely, automatically, and efficiently completes large-scale component installation [90]. This indicates that digital twins are not merely “visual interfaces” but can serve as operating environments for trajectory planning and control.

In the aircraft fuselage section docking scenario, Kang et al. constructed a trial assembly simulation and accuracy assurance system based on dynamic process models [104]. Its core innovation is integrating digital measurement, NC positioning, and virtual trial assembly into a unified process chain, making the originally “invisible and unmeasurable” insertion end docking process digitally visible [104]. Through modules of point cloud data acquisition, pose solving, control integration, and virtual simulation, the system can perform digital trial assembly before actual docking, reducing repeated trial assembly and degraded connection consistency [104]. The practical significance of such methods is obvious, as rework costs for interference or uneven gaps in large fuselage section docking are often far higher than conducting a high-quality virtual trial assembly in advance.

Kang further proposed an accurate docking algorithm for typical barrel segments driven by real-time perception data [91]. Its core idea is not static simulation but continuously updating the digital twin model based on real-time measurements and iteratively simulating the next assembly action in virtual space until precise alignment is achieved [91]. This step-by-step iterative mechanism of “measure once, simulate one step, execute one step” is the key difference between digital twin control and traditional offline simulation. It transforms trajectory planning from a one-time solving problem into an online decision problem continuously corrected with the assembly process.

In scenarios emphasizing process monitoring and comprehensive quality control, digital twins also excel in multi-physics and multi-source error coupling modeling. Mei et al. proposed a physically simulation-enhanced pose adjustment system in large-scale aircraft wing box assembly, incorporating temperature fluctuations and gravity effects into the pose alignment model through finite element analysis [106]. Compared with classic best-fit-driven digital pose adjustment systems, this method significantly reduces deviations of key feature points and has been applied to large-scale aircraft wing box assembly at AVIC [106]. This indicates that the true contribution of digital twins to large-scale component assembly is not only path planning but identifying “why the same path fails to achieve expected results in real environments”.

Fan proposed a digital twin adaptive alignment system for large-scale cylindrical aerospace components, unifying geometric, physical, functional, and data models into a DT process system, and realizing adaptive force-position control by combining measured poses, alignment stresses, and predicted errors [92]. The core innovation of this study is placing real-time state perception, error prediction, and process execution decision-making into the same twin closed loop, enabling the system to know not only “current deviation” but also “how to adjust next and what consequences will result” [92]. Ma’s digital twin quality online prediction and control framework for large-scale solid rocket motor assembly reflects the same trend: advancing assembly quality prediction to the process execution stage through point clouds, deviation tracing, and intelligent adjustment mechanisms [105].

Overall, the core value of digital twin technology is to explicitly represent partially invisible states, future risks, and multi-source deviation propagation processes in large-scale component assembly [90,91,106]. Its advantages are rich constraint expression, strong process interpretability, and easy integration of quality prediction and virtual trial assembly; however, critically, current digital twins are still far from serving as a reliable primary basis for online decision-making.

First, model fidelity remains the core constraint. Thermal deformation, gravity deflection, clamping boundary changes, and multi-source error coupling in large-scale component docking assembly continuously alter real system states, and Mei precisely showed that even if the pose adjustment system itself is accuracy-verified, deviations of key feature points still struggle to converge stably without explicitly incorporating temperature fluctuations and gravity effects into the model [106]. This means many digital twin systems lack not geometric models but sufficiently credible physical and error propagation models. If twin models cannot stably characterize these dominant factors, their online prediction results cannot serve as direct bases for high-risk assembly decisions.

Second, online decision-making heavily relies on high-quality real-time perception, which is one of the weakest links in large-scale component assembly. Both Kang and Kang regarded “invisible and unmeasurable docking ends” as the direct motivation for introducing digital twins [91,104]. Conversely, this means reliable digital twin-driven online decision-making requires continuous access to sufficiently complete and delay-controllable measurement updates; otherwise, optimal decisions in the virtual space may be based on outdated or partially missing states. Ma further integrating high-fidelity modeling, deviation tracing, and online quality prediction into a unified framework also reflects that digital twin effectiveness highly depends on synchronized closure of measurement, data, and model chains [105].

Third, current digital twin methods excel at offline trial assembly and iterative optimization but face computational and synchronization pressures under strict real-time constraints. Meng successfully completed automatic large-scale component installation via digital twin + PRM, and Kang achieved accurate barrel segment docking through the iterative “measure-simulate-execute” approach [90,91]. These studies prove digital twin feasibility but also show that the mainstream working mode remains “online iterative correction” rather than assuming primary control laws in high-frequency closed loops like underlying servo control. As model complexity rises to include contact, thermal effects, flexible deformation, and multi-sensor fusion, time budgets for cyber-physical synchronization and online solving tighten rapidly [104,105].

Fourth, verification levels limit the persuasiveness of digital twins as reliable decision bases. Most existing representative studies have completed experimental, scaled, or single-scenario engineering verifications—such as Meng’s robot automatic assembly experiment, Kang’s MA700 front-middle fuselage scaled test environment and typical barrel segment docking experiment, and Fan’s application verification [90,91,92,104]. These achievements demonstrate significant digital twin potential but still fall short of industrial-grade reliability for “stable cross-model, cross-workshop, and cross-environment migration”. Liu’s digital twin control framework for full-life-cycle quality data also indirectly indicates that digital twins can hardly form a long-term stable decision basis without continuous, standardized, and traceable quality data governance [115].

Therefore, a more prudent conclusion is that current digital twins are best suited for high-value process visualization, offline trial assembly, error prediction, and low-to-medium frequency strategy optimization, rather than being simply interpreted as an “all-round decision core” that can independently replace highly reliable online controllers [90,104,105]. In the foreseeable stage, the practical implementable system will more likely use digital twins for state reconstruction, risk prediction, and next-step action suggestions, with multi-sensor fusion and traditional control loops responsible for high-frequency execution and safety constraint bottoming [91,92,115].

### 5.4. Flexible Assembly Technology

Flexible assembly technology is one of the most critical and challenging directions in trajectory planning and control for docking assembly of large-scale components. The fundamental reason is that many large-scale aerospace components—especially composite wall panels, fuselage sections, airfoils, and thin-walled frames—do not satisfy the rigid body assumption during assembly. Components undergo significant shape changes under gravity, clamping, transportation, local contact, and actuator loading, which in turn alter feasible trajectories, contact sequences, gap distributions, and required control forces [10,114]. Therefore, the true task of flexible assembly technology is not only to make the control system “more robust to deformation” but to incorporate deformation itself into planning objects, expanding control objectives from pose alignment to shape alignment and gap alignment.

Saadat systematically analyzed deformation during assembly of large-scale aerospace components, revealing non-negligible shape changes of components under self-weight and assembly loads [114]. The significance of such studies is mechanistically explaining why rigid-body-assumption-based trajectory planning often fails on-site. If the control system only tracks nominal component poses rather than real surfaces, local contact and gaps may still severely exceed tolerances even with small theoretical pose errors [111,114].

A key prerequisite for flexible assembly to evolve from empirical compensation to systematic planning is the development of flexible body modeling methods. Currently, flexible body modeling for trajectory planning and control of large-scale component assembly mainly develops along three interrelated routes: reduced-order finite element models, floating frame of reference formulation, and component mode synthesis.

Table 6 summarizes three representative flexible body modeling methods and their significance in assembly planning.

Reduced-order finite element methods mark the first step for flexible assembly modeling to enter the control loop. Nowakowski et al. pointed out that finite element discretization introduces numerous elastic DOFs in elastic multi-body systems, and efficient simulation relies on linear order reduction of these DOFs [107]. Their study systematically compared modern reduction techniques and emphasized the importance of structure-preserving reduction in flexible multi-body systems [107]. This is directly relevant to large-scale component assembly control, as oversized models prevent trajectory optimization and online control from executing within industrial takt times. Sonneville et al. further summarized mode reduction procedures in flexible multi-body dynamics, showing that reduction is not simply deleting high-order modes but selecting retained modes based on target frequency bands, interface responses, and observation-control requirements [116]. In other words, the quality of reduced-order models determines not only simulation speed but also whether the controller can “see” deformation modes truly affecting assembly quality.

The floating frame of reference formulation is one of the core theoretical foundations for flexible assembly modeling of large-scale components. The floating frame of reference idea early proposed by Canavin and Likins allows decomposing the total motion of a flexible body into rigid-body motion of the floating frame and small elastic deformation relative to that frame [108]. This idea is particularly suitable for aerospace large-scale component assembly, as components typically undergo large overall pose changes with relatively small but non-negligible local elastic deformations. Bauchau and Rodriguez proposed a mode-based element formulation in nonlinear flexible multi-body dynamics, clearly showing that significant computational cost reduction can be achieved via mode expansion under the floating frame of the reference framework, and the model strictly degenerates to rigid-body motion equations in the absence of elastic deformation [117]. This property is critical, as it ensures flexible models can be naturally embedded into existing rigid-body control frameworks without completely overturning established kinematic solving and control architectures.

In large-scale complex component systems, the floating frame of reference formulation alone is insufficient; interface coupling and model scale issues must be further addressed, which is where component mode synthesis plays a role. Spanos and Tsuha systematically discussed mode selection in flexible multi-body simulation, noting that selection of different mode sets such as Craig–Bampton, MacNeal–Rubin, and Benfield–Hruda significantly affects the retention of input–output responses in the frequency band of interest [119]. Cammarata and Pappalardo adapted classic structural dynamics reduction methods such as Guyan-Iron condensation, mode truncation, and Craig–Bampton synthesis to the FE–FFRF framework, with numerical experiments showing consistent and correct flexible multi-body reduction results for all three methods [109]. The core innovation is not only “proving these methods work” but clarifying the coupling relationship between reference conditions, interface conditions, and mode selection in flexible multi-body systems [109]. Cammarata further discussed interface reduction, showing that interface DOF handling directly affects computational efficiency and contact response accuracy for multi-body systems [118].

These flexible body modeling methods only demonstrate engineering value when truly integrated into assembly planning and control. Park and Mills combined finite element modeling, component mode synthesis, and reduction methods in flexible load assembly research for shape control and vibration suppression during gripper operation of thin-walled parts [120,121]. Park explicitly noted that flexible thin-walled parts experience both static shape deviations due to gravity and vibrations due to inertia during robot assembly, requiring control systems to simultaneously handle reshaping and vibration damping [121]. The core innovation is integrally modeling the clamping system and workpiece system based on flexible models, then designing shape and vibration controllers via reduced-order models [121]. Although not directly targeting large aircraft component docking, these studies provide key methodology for connecting flexible body models to closed-loop control.

In aviation assembly applications, Jayaweera’s adaptive robot assembly research marks the entry of flexible assembly into engineering control [10]. The key idea is no longer treating compliant aerostructure components as “error-causing objects” but regarding their compliance as a system attribute that can be sensed, utilized and controlled [10]. This shifts control strategies from rigid-body alignment to adaptive assembly based on component responses. Stolt and Jonsson further conducted force-controlled assembly research on flexible aircraft structures and flexible ribs, reflecting the same trend: the final control goal is no longer merely guiding the end-effector to a target position but guiding flexible components to a target contact state [101,122].

As composite fuselage assembly issues become more prominent, flexible assembly technology has expanded from “contact control” to “shape control”. Mou et al. proposed a sparse sensor placement-based adaptive control method (SPAC) for high-precision fuselage assembly [11]. The core innovation is acknowledging deviations in mechanical properties between design models and actual incoming fuselages, thus updating models online via sparse sensor feedback instead of assuming nominal models remain accurate [11]. This work has strong engineering value, as full-field high-precision online measurement of large fuselage surfaces is expensive and difficult to implement, while sparse sensing significantly improves control performance at acceptable measurement costs [11].

Du et al. further pointed out that existing practices often adjust each incoming component to the design shape, which may not be the optimal strategy for paired assembly [123]. Their proposed sparse learning model directly targets maximum gap between two components for shape control and actuator position selection, shifting the control goal from “approximating nominal shape” to “minimizing real assembly gaps” [123]. This shift is critical, indicating that flexible assembly control now truly targets assembly quality rather than upstream design benchmarks. Manohar et al. used machine learning and sparse sensing to predict shim gaps, also reflecting the extension from geometric measurement to quality prediction [94].

At the control level, Wang proposed a coordinated force and shape control method for large-scale composite fuselage wall panel assembly [12]. The significance of this technical route is no longer treating contact force control and shape adjustment as independent problems but unifying them into the same assembly loop. This is critical for large-scale composite wall panels, as contact force distribution alters local shapes, which in turn change subsequent contact force distributions—they are inherently coupled. Ari17 proposed flexible best fit assembly in an Airbus A350 XWB case(Rolls-Royce Holdings plc, Derby, UK), reflecting the same logic: seeking a globally better balance between component local deviations and assembly interface requirements via flexible best fitting [111].

In recent years, flexible assembly technology has further integrated with data-driven and reinforcement learning methods. Lutz et al. proposed a model-free reinforcement learning method for fuselage shape control, training control strategies in a finite element simulation environment to directly output shape adjustment actions based on different incoming states [93]. Case studies show that this method reduces the root-mean-square gap between components by an average of 98.4% compared with initial shape mismatch, achieving smaller final shape errors, lower maximum actuation forces, and lower sample-to-sample fluctuations than baseline methods [93]. This indicates that flexible assembly control strategies are expanding from “explicit model-based solving” to “model-trained strategy execution”, premised on reasonable representation of flexible response mechanisms.

Another important application route of flexible body modeling in assembly planning is constructing sufficiently fast and accurate equivalent mechanical models to support multiple iterative optimizations. Qu proposed an efficient equivalent mechanical model for composite wall panel assembly and applied it to assembly process optimization [110]. The core engineering significance of such work is replacing high-dimensional finite element models originally difficult to integrate into optimization loops with low-dimensional surrogate models callable repeatedly within reasonable time, making “deformation-aware trajectory optimization” practically usable [110].

Overall, planning and control based on flexible assembly technology have completed three levels of transitions: first, from treating component deformation as disturbance to regarding deformation as a state variable [10,114]; second, from relying solely on empirical compensation to computable models based on reduced-order finite elements, floating frame of reference formulation, and component mode synthesis [107,108,109]; third, from pose-oriented control to joint optimization of gaps, shapes, and contact quality [12,93,123]. Current bottlenecks mainly lie in model-physical consistency, online observation costs, and computational speed; however, from a development trend, flexible assembly is likely to become the core control framework for automated assembly of large-scale composite structures.

### 5.5. Other New Technologies

In addition to the four main routes mentioned above, trajectory planning and control in the docking assembly of large-scale components are rapidly absorbing a batch of newer technologies that are more “quality-oriented” and “decision-oriented”. A common feature of these technologies is not merely improving trajectory solving speed, but changing the way the control problem itself is defined—specifically, moving gaps, shimming, deformation, process costs, and task constraints that were originally addressed in later stages to the planning stage for unified consideration. Based on current literature development, machine learning-driven gap prediction, constraint programming, and task-level programming, as well as single-step shimming and virtual shimming technologies, have emerged as the most active growth lines in this direction [94,95,96,110,124]. Compared with the traditional control, digital twins, and compliant assembly emphasized earlier, these new technologies more clearly reflect a trend: trajectory planning is shifting from “how to move” to “how to achieve acceptable assembly quality with lower risk and cost”.

Machine learning first transforms the position of assembly quality prediction in the control chain. Manohar et al. proposed a gap prediction method for aircraft assembly using historical production data and sparse sensing, enabling the system to predict shim gap distribution before actual docking, thereby reducing reliance on full-scale measurement and repeated trial assembly [94]. Subsequently, the research focus further expanded from “predicting existing gaps” to “predicting how deformations and gaps evolve with control actions”. For example, Yan et al. studied the prediction of gap volume for wing assembly [125], Qi et al. constructed a combined prediction model for aircraft panel assembly deformation [126], and Sun et al. explicitly incorporated structural deformation factors into gap prediction, using generative surrogate models to enhance the expression ability for complex distributions [127]. The common significance of such work is that they transform assembly evaluation problems originally relying on post hoc inspection into a priori prediction problems that can be embedded into the planning process. Correspondingly, the optimization object of the controller is no longer merely the target pose error, but the joint result of the final gap distribution, subsequent compensation requirements, and process risks. However, the main limitation of this route is clear: model generalization ability usually depends on the coverage of training samples, and transferability to new models, new boundary conditions, and abnormal working conditions remains insufficient [94,126,127].

Constraint programming and task-level programming reshape the trajectory planning problem from another perspective. The constraint-based robotic assembly programming method proposed by Halt et al. shows that complex assembly tasks can no longer rely on a large amount of low-level trajectory code, but can be described through geometric constraints, contact conditions, and event-triggered rules [96]. Polverini et al. further applied constraint-based skill programming to lightweight collaborative robot assembly, demonstrating the ability of reactive trajectory generation based on force control requirements [128]. Arbo et al. directly introduced CAD information into the constraint-based assembly system architecture, forming a more coherent chain between task description, process-layer reasoning, and control-layer execution [129]. At a more macro level, Kardos et al. proposed a constraint model for assembly planning, combining macro process planning with micro process feasibility feedback [130]. The common value of these studies is their attempt to transform process knowledge from empirical programming into computable constraints, thereby reducing reprogramming costs under conditions of high-mix, low-volume production and frequent model changes. This is particularly critical for large-scale component assembly, as the real bottleneck often lies not in whether a single trajectory is solvable, but in whether complex contact processes and process rules can be converted into stable, reusable executable control logic. However, this direction is currently more mature in skill verification for small-to-medium-scale assembly, and its coverage of large-scale flexible components, multi-sensor closed loops, and complex compensation processes remains insufficient [96,128,130].

Single-step shimming and its related digital compensation technologies directly reincorporate post-assembly compensation problems into the planning stage. The core of the single-step shimming scheme proposed by Ehmke et al. is not merely simplifying the shimming process, but attempting to compress the traditional compensation cycle—highly dependent on the “measurement-trial assembly-disassembly-machining-reassembly” loop—into a more forward-leaning and continuous process [95]. Wang et al. studied shimming design and optimal selection for non-uniform gaps in wing assembly, realizing hybrid shim scheme design through scan data, point cloud local region extraction, and finite element evaluation [131], indicating that shimming problems are shifting from empirical fitting to data-driven and model-driven joint decision-making. Esposito et al. further proposed physics-driven digital twin-based virtual shimming simulation, combining online measurement with physical simulation to significantly reduce repeated measurement and trial assembly cycles in skin assembly [132]. From the perspective of planning and control, the importance of such technologies is often underestimated, as they actually change the boundaries of the control problem: the control objective is no longer limited to delivering components to the target pose, but also includes considering whether subsequent shims are needed, how shims should be designed, and how the compensation process can be minimized. For this reason, single-step shimming technology has a natural coupling relationship with gap prediction, deformation surrogate models, and digital twins [95,131,132].

In addition, a number of technologies between model-driven and pure learning are rapidly entering this field. Yan et al. applied Gaussian processes considering uncertainty to compliant assembly control of complex-shaped composite components, enabling predictive models to provide both estimated values and uncertainty information [112]. Zhang et al. proposed a conditional autoencoder-based surrogate modeling framework for assembly deformation [124], and Qu et al. constructed an efficient equivalent mechanical model applicable to composite panel assembly optimization [110]. Furthermore, Bayesian optimization methods for online optimization have begun to be used for shape adjustment of large composite fuselage panels under individual difference conditions [133], and even quantum Bayesian optimization frameworks attempting to improve sample efficiency have emerged [134]. This indicates that the most active new technologies currently are not merely “learning controllers”, but are more broadly reshaping the prediction, optimization, constraint expression, and process compensation links in assembly control.

Overall, these new technologies collectively drive trajectory planning and control in the docking assembly of large-scale components from a “trajectory generation problem” to a “joint decision-making problem of quality-risk-efficiency” [94,95,96,124]. Among them, machine learning-based gap prediction changes the timing of quality information entering the control chain, constraint programming reduces the threshold for converting complex assembly knowledge into control programs, and single-step shimming technology reincorporates later compensation processes into earlier planning. In the future, it is more likely that these methods will not replace each other, but will further couple with digital twins, compliant body modeling, multi-sensor fusion, and traditional force-position control to form a unified planning and control framework targeting gaps, shape, contact forces, and compensation costs as common objectives [110,112,124,133].

## 6. Conclusions and Prospects

This paper systematically reviews relevant research on automated assembly of large-scale aerospace components from three dimensions: pose adjustment and positioning mechanisms, digital measurement technologies, and trajectory planning and control. Synthesizing existing literature shows that technological evolution in this field does not follow the improvement of single equipment performance but progresses toward “reconfigurable mechanisms, online measurement, closed-loop control, flexible modeling, and digital decision-making” [1,2,5]. What truly drives the engineering practicality of automated assembly of large-scale components is no longer an isolated technology but the collaborative coupling of execution, perception, and decision-making capabilities under a unified coordinate benchmark and unified quality objectives.

From the overall current status, the field has completed three important transformations: first, the source of assembly precision is shifting from heavy fixed tooling to reconfigurable mechanisms and online metrological closed loops [3,19,21]; second, the state description of the assembly process is shifting from a small number of discrete point measurements to multi-source sensing, point clouds, and process-level uncertainty representation [4,8,50]; third, control objectives are shifting from “moving components to target poses” to “achieving high-quality docking under joint constraints of deformation, gaps, contact forces, and quality risks” [12,93,97]. In other words, automated assembly of large-scale aerospace components is evolving from a rigid geometric problem into a multi-physics, multi-source information, and multi-layer control coupled problem.

At the same time, existing research shows obvious structural fragmentation. Mechanism research often emphasizes load-bearing, stiffness, and reconfiguration capabilities but rarely takes measurement uncertainty and control observability as mechanism design variables [13,25]. Measurement research can deeply analyze error sources and propagation laws but mostly stays at the metrological evaluation level, not fully entering real-time trajectory planning and online controller design [8,9,75]. Planning and control research has begun to consider multi-sensor fusion, digital twins, and flexible assembly, but many works still rely on local task optimality, specific test platform verification, or simplified model assumptions [11,88,91]. Therefore, the core current issue is no longer “whether enough methods exist” but “whether these methods are organized into a unified system supporting industrial-grade high-reliability closed-loop assembly”.

### 6.1. Integrated Field Judgments

Based on this review, the following integrated judgments can be formed.

First, future competitive automated assembly systems for large-scale components will not be dominated by a single type of equipment but feature heterogeneous system collaboration. NC positioners, parallel mechanisms, industrial robots, and new mobile mechanisms will assume distinct roles such as high-load support, multi-DOF docking, large-range process execution, and constrained space operation [21,24,29,35]. Correspondingly, laser trackers, iGPS, vision systems, and scanning systems will not replace each other but jointly form a hierarchical measurement system [4,50,53,60]. The superiority of future systems will increasingly depend not on standalone performance but on the interoperability of heterogeneous units under a unified reference frame and unified quality evaluation criteria.

Second, the concept of “precision” in large-scale component assembly is changing. Historically, precision was mainly understood as position or pose error; at the current research frontier, precision increasingly reflects a comprehensive result of gap distribution, contact state, shape consistency, and quality risk [12,97,111]. This means subsequent studies still taking pose error as the single objective function can hardly truly represent engineering assembly quality.

Third, measurement uncertainty will shift from a metrological auxiliary issue to a first-class variable in planning and control. Existing studies fully show that error sources in large-scale assembly include not only sensor noise but also reference network geometry, environmental disturbances, calibration residuals, and data fusion processes [8,50,75,77]. Therefore, future high-reliability assembly systems must shift from “control after measurement” to “control with uncertainty”.

Fourth, flexible assembly is not an auxiliary issue in the composite era but one of the main problems in large-scale component assembly control. As the proportion of thin-walled parts, composite wall panels, and large-scale flexible shell segments increases, the rigid body assumption is only suitable as a local approximation and can no longer serve as the full-process main model [10,11,93,114]. Therefore, flexible body modeling, shape control, and contact quality optimization should no longer be regarded as specialized issues but integrated into the general framework of docking assembly control for large-scale components.

### 6.2. Verifiable Core Research Gaps

Unlike general “worthy of research” statements, Table 7 presents key, verifiable research gaps clearly identified based on the literature covered in this paper. “Not yet seen” herein means no corresponding results have been found in the representative public literature systematically reviewed in this paper.

Among the above gaps, the first and third are priorities for breakthrough. Gap 1 and Gap 3 are prioritized over others primarily, because they correspond to the two most fundamental cognitive premises in automated assembly of large-scale components: “whether current measurement results are credible” and “how components will respond during motion and contact”. The former determines if the controller can correctly understand its own perception boundaries, and the latter ensures the controller can accurately predict the true behavior of the controlled object [8,9,12,50,107,109]. Without stable modeling and integration of these two premises into closed-loop planning, subsequent higher-level capabilities—such as digital twin credibility management, joint optimization of gaps and contact forces, and data-driven strategy transfer—will only be built on an unstable foundation.

In contrast, while Gaps 2, 4, 5, and 6 are equally important, they logically depend more on breakthroughs in the first two. Decision-level credibility management of digital twins essentially relies on quantifiable uncertainty propagation and updatable component response models; otherwise, “credibility” remains conceptual and cannot enter control decisions [91,105,106]. Integrated optimization of gaps, contact forces, shape errors, and compensation processes must also be based on sufficiently accurate descriptions of flexible deformation and contact evolution; otherwise, unified solving can only achieve formal coupling rather than practically executable process solutions [12,95,97]. As for industrial verification benchmarks and data-driven method transferability, they act more as amplifiers and verifiers for mature systems rather than foundational conditions determining the system’s feasibility [92,93,94,102].

Thus, from a research perspective, prioritizing Gaps 1 and 3 is not merely because these issues are more “cutting-edge”, but because they form the shortest bottleneck for the field to advance from local effectiveness to systematic reliability. Only when multi-sensor uncertainty can be integrated into real-time closed-loop planning and flexible body models can truly participate in online docking solving can automated assembly of large-scale components move beyond “measurable, controllable, and optimizable” to “verifiable, reproducible, and scalable” [1,2,11,75]. In this sense, higher priority does not negate the importance of other gaps but reflects judgments on technical dependencies and system evolution sequences.

### 6.3. Research Routes for the Next Stage

Based on the above judgments, future research should advance in the following directions:

First, shift from “point-to-point technology integration” to “unified constraint modeling”. Mechanism constraints, measurement uncertainty, contact force boundaries, flexible deformation, and quality criteria in future assembly systems need to be uniformly written into the same planning and control problem instead of being handled in segments by different modules [12,50,97]. Only then can the system truly move from local optimality to process optimality.

Second, shift from “static model-driven” to “credible online model-driven”. Both digital twins and flexible body models should not only be used for offline analysis but possess the ability to continuously correct with measured data [91,106,110]. The key here is not only online updating itself but establishing a model credibility management mechanism, allowing the controller to know when to trust the model and when to revert to conservative control strategies.

Third, shift from “high-precision measurement supporting control” to “control with uncertainty”. Future high-value research should not stop at improving measurement precision itself but further study how to directly integrate measurement uncertainty, fusion covariance, and environmental disturbance boundaries into trajectory planning, termination criteria, and safety constraints [8,50,75,81]. This will be a key step for automated assembly of large-scale components to evolve from “operable automatically” to “certifiable, reproducible, and releasable”.

Fourth, shift from “rigid trajectory planning” to “quality-oriented flexible process control”. For large-scale composite structures and thin-walled components, future controller objective functions should no longer be limited to pose errors but directly target gaps, shape consistency, peak contact forces, subsequent compensation amounts, and connection quality [12,93,123]. This means boundaries between trajectory planning, shape control, and process compensation will become increasingly blurred.

Fifth, strengthen reproducible experimental platforms and evaluation benchmarks. Many current studies are difficult to compare horizontally not due to unclear method merits but due to large differences in verification tasks, measurement conditions, and evaluation indicators [92,102,104]. Without unified benchmark problems, quality indicators, and uncertainty reporting methods, the field will struggle to form truly cumulative research progress.

### 6.4. Concluding Remarks

Overall, automated assembly of large-scale aerospace components is entering a stage where competitiveness is determined by system-level capabilities. The most important future breakthroughs in this field are unlikely to come from further extreme optimization of a single point technology but from the collaborative maturity of four capabilities: high-adaptability execution represented by reconfigurable mechanisms, high-credible perception represented by large-scale digital metrology, high-quality decision-making represented by uncertainty awareness and digital twins, and high-fidelity process control represented by flexible body modeling and shape control [2,50,93,106]. Only when these four capabilities are truly unified in the same closed loop can automated assembly of large-scale aerospace components evolve from “superposition of several advanced technologies” to an “industrial-grade system deployable at scale”.

## Figures and Tables

**Table 1:** Comparison of four pose adjustment and positioning mechanisms [13,18,19,20,21,22,23,24,25,26,27,28,29,30,31,32,33,34,35,36].

Technical Category	NC Positioner	PKM	Industrial Robot	Other Novel Mechanisms
**Load-Bearing Capacity**	High [13,18,19]	Medium to high [23,24,25]	Medium; dependent on body specification and end process head [28,29]	Low to medium; expandable for some mobile heavy-duty systems [33,34,35]
**Workspace**	Medium to large; depending on frame scale and support point layout [20,21]	Medium; constrained by singular configurations and mechanism topology [26,27]	Large [28,30]	Very large or unique advantages in constrained spaces [33,34]
**Representative Positioning Accuracy**	The absolute accuracy of on-site pose adjustment is approximately ±0.05 mm [21]	The literature generally reports high-precision pose adjustment capabilities, but no unified absolute value index [23,24,25].	Hole positions meet ±0.060 in specifications [28], accuracy on parts is better than ±0.25 mm [29], and the positioning of drilling and riveting unit is approximately 0.05 mm [31].	Most focused on task accessibility and system demonstration verification; the Autonomous Industrial Mobile Manipulator (AIMM) scheme has completed 1:1 scale verification [35].
**Stiffness**	High [13,19]	High [23,24]	Medium to low; insufficient body stiffness requiring compensation [30,32]	Varying greatly by mechanism, usually compensated by end reshaping or special structures [35,36]
**Cost**	Medium to high; high initial investment but good reconfiguration reusability [18,20]	High [3,25]	Medium; mature equipment and strong scalability [15,29]	Medium to high, complex system integration [17,36]
**Typical Applications**	Wing and wing-box assembly, flexible fixtures, datum support, key characteristic realization [19,22]	Wing and fuselage section docking, wing-box front spar and rib installation, drilling and riveting collaborative support [24,25]	Automatic drilling, riveting, hole-making, hole position correction, large-range pose fine-tuning [28,30,31]	Internal wing-box operations, mobile assembly, flexible fuselage wall panel final assembly, fixed-station-free scenarios [33,36]
**Technological Maturity**	High [13,19,21]	Medium to high [24,25]	High [28,29,31]	Medium [33,35,36]

**Table 2:** Comparison of representative digital measurement technologies [4,48,52,53,54,55,56,57,58,59,60,61,62,63,64,65,66,67,68,69,70,71].

Technical Category	Multi-Camera and Vision Measurement	Laser Tracker	iGPS	Laser Scanning and LiDAR	Portable Photogrammetry and Structured Light Hybrid Scheme
**Measurement Range**	Typical large-field-of-view systems can cover approximately 4 × 3 × 2 m^3^, expandable by multi-camera vision fields [55,56]	Suitable for large-volume measurement scenarios larger than 10 m [4,48]	Test volume of approximately 10 m × 10 m × 2 m and measurable length up to 12 m, with scalable network potential [47,60,64]	Capable of acquiring dense point clouds of large curved surfaces and complex components [53,54]	Used in large-volume scenarios of approximately 3 m to 64 m [52]
**Accuracy**	Depth camera systems can achieve approximately 0.05° pose angle accuracy [57].	Multi-laser-tracker reference networks can achieve approximately 10 µm-level expanded uncertainty of reference coordinates in a volume of approximately 10 m × 10 m × 2 m [60].	Length uncertainty: approximately 170 µm; coordinate uncertainty: approximately 120 µm × 120 µm × 190 µm; k = 2 [60]	Point cloud registration can reach a sub-millimeter level, deviation between the template hole center and laser tracker reference result is within 0.2 mm [53]	Accuracy is significantly affected by camera layout, triangular intersection geometry, and operator skills [52,70].
**Anti-occlusion Capability**	Medium to high, multi-view layout alleviates single-line-of-sight occlusion [56,58]	Low, strongly dependent on line-of-sight conditions [4,46]	Medium to high, network layout is superior to single-station equipment [47,64]	Medium, multi-station scanning improves occlusion but depends on registration quality [53,69]	Medium [52,71]
**Cost**	Low to medium, easier to promote than high-end large-scale single-point equipment [52,56]	High [4,61]	High, requiring deployment of transmitter networks [47]	Medium to high [53,54]	Low to medium [52]
**Update Frequency**	High, suitable for online pose update [57,59]	Medium [4]	High, suitable for dynamic tracking and online feedback [65,66]	Medium to high, suitable for rapid surface acquisition [53,54]	Medium to high [52,71]
**Uncertainty Analyzability**	Medium, greatly affected by calibration model, feature extraction, and reconstruction algorithms [52,55]	High, with ISO 10360 extended verification ideas and ASME B89.4.19 system [61,62,63]	Medium, system configuration and environment significantly affect results [47,67,68]	Medium to low, registration parameters and software processing significantly affect results [69]	Medium, suitable for process-level evaluation combined with Monte Carlo [52]
**Technological Maturity**	Medium to high [57,58,59]	High [4,61]	Medium to high [47,60,68]	Medium [53,54,69]	Medium [52,70,71]

**Table 3:** Primary uncertainty sources and propagation paths in large-scale component digital measurement (→ means “to”).

Uncertainty Source	Typical Manifestations	Main Propagation Paths	Engineering Impacts	Representative Studies
Sensor intrinsic errors	Range noise, angular encoding errors, axis eccentricity, opto-mechanical misalignment [63,77]	Raw observations → 3D coordinate solution	Systematic coordinate bias and length errors	[61,63,77]
Calibration residuals and incomplete modeling	Unmodeled high-order camera distortion, hand-eye calibration residuals, equipment geometric parameter errors [49,55]	Equipment coordinates → unified coordinate system transformation	Systematic bias introduced into the entire process	[49,55,78]
Environmental factors	Thermal drift, air turbulence, humidity and refractive index changes, workpiece thermal expansion [51,61,79]	Simultaneous changes in sensor readings and true workpiece geometry	Degraded long-term measurement stability, discrepancies between on-site and laboratory results	[51,61,79]
Network geometry and reference point configuration	ERS fails to envelope critical areas, unreasonable station layout [74,75]	Multi-station stitching and coordinate transformation	Amplified errors in critical regions	[49,74,75]
Multi-sensor fusion and registration	Sensitivity of point cloud registration parameters, coupling of local and global sensor covariances [50,69,80]	Local measurements → global pose and gap calculation	Results sensitive to algorithm and software parameters	[50,69,80]
Measurement strategy and operator factors	Sampling paths, feature point selection, camera layout, operator experience [9,52]	Observation scheme → final evaluation value	Obvious result differences for the same equipment under different workflows	[9,52]

**Table 4:** Characteristics of typical uncertainty quantification frameworks.

Framework	Core Idea	Advantages	Limitations	Applicable Scenarios	Representative Studies
GUM and GUF analytical propagation	Propagating uncertainty based on sensitivity coefficients and covariance matrices [82,85]	Computationally efficient; results easily integrated into filtering, registration, and system design [50,75]	Sensitive to strong nonlinearity, non-Gaussian distributions, and complex constraints [9,81]	Coordinate transformation, multi-station network design, online fusion	[50,75]
Monte Carlo propagation	Directly propagating input distributions via random sampling [9,83]	Suitable for complex models, no explicit partial differentiation required, handling sampling strategy effects [9,52]	High computational cost, limited online application	Task-specific uncertainty, complex point cloud and vision workflows, process design phase	[9,52,83]
Hybrid and task-specific frameworks	Combining analytical propagation with simulation verification, or customized for virtual assembly and multi-sensor fusion	Balancing engineering efficiency and model fidelity [75,81]	Complex implementation, dependent on scenario-specific modeling quality	Virtual assembly, multi-sensor fusion, process planning optimization	[50,75,81]

**Table 5:** Comparison of five trajectory planning and control technologies [86,87,88,89,90,91,92,93,94,95,96,97,98,99,100,101,102,103,104,105,106,107,108,109,110,111,112].

Technical Category	Traditional Trajectory Planning and Control	Multi-Sensor Fusion Control	Digital Twin-Driven Control	Flexible Assembly Technology	Other New Technologies
**Constraint Handling Capability**	Medium to high; exceling in pose, contact, and local force constraints [86,97,98]	High; handling measurement, stating estimation, and contacting constraints simultaneously [88,89]	High; uniformly handling collision, process, pose, and quality constraints [90,91,92]	High; handling contact, gaps, shape control, and compliance constraints [10,12,93]	Medium to high; depending on learning or task programming frameworks [93,96]
**Real-Time Performance**	Medium to high; suitable for online execution but relying on simplified models [87,99]	Medium; affected by fusion algorithms and measurement update frequency [88]	Medium; strong offline simulation, online capability depends on model update speed [104,105]	Low to medium; constrained by flexible model scale and online observation capability [11,107]	Medium to high; fast online execution after training [93]
**Deformation Modeling Capability**	Low to medium; usually approximating components as rigid or locally compliant bodies [100,101]	Medium; indirectly reflecting deformation and pose deviations via measurement feedback [88,103]	Medium to high; incorporating thermal deformation, gravity, and process deviations into cyber-physical closed loops [92,106]	High; core advantage [108,109,110]	Medium to high; relying on surrogate models or data-driven representation [94,112]
**Verification Level**	Laboratory to industrial application [98,102]	Laboratory to engineering verification [88,89]	Laboratory to industrial verification [90,91,106]	Simulation to industrial cases [10,11,111]	Simulation to laboratory, small-scale engineering trials [93,95]
**Technological Maturity**	High [97,98,99,100]	Medium to high [88,89,103]	Medium [91,92,104]	Medium [108,109,110]	Medium [93,94,112]

**Table 6:** Three typical flexible-body modeling methods and their applications in assembly planning.

Modeling Method	Core Idea	Main Advantages	Main Limitations	Relationship to Assembly Planning and Control
Reduced-order finite element	Extracting dominant modes or low-dimensional states from high-dimensional finite element models [107,116]	Significantly reducing computational load while retaining main elastic features [107]	Reduction quality highly dependent on mode selection and working condition matching [116]	Enabling flexible body models to enter online optimization and closed-loop control
Floating frame of reference formulation	Separately modeling large rigid-body motion and small elastic deformation [107,108]	Suitable for assembly processes with large displacements and small deformations [108,117]	Complex modeling under complex interfaces and strong nonlinear contact [118]	Providing a unified framework for pose changes and local elastic coupling of large-scale components
Component mode synthesis	Representing flexible components with combinations of fixed-interface, free-interface, or constrained modes [109,117,119]	Balancing accuracy and efficiency, and facilitating multi-component coupling [109]	Improper mode and interface condition selection significantly affects results [109,119]	Suitable for rapid solving of multi-component, multi-fixture, multi-contact point assembly systems

**Table 7:** Verifiable core research gaps in automated assembly of large-scale aerospace components.

Research Gap	Existing Progress	Key Unresolved Issues	Verifiable Target Form
Disconnection between multi-sensor uncertainty propagation and closed-loop planning	Multi-sensor fusion docking [88], GUM-compliant covariance fusion [50], task-specific uncertainty analysis [9,81]	No real-time closed-loop trajectory planner explicitly integrating GUM-compliant heterogeneous sensor fusion uncertainty transmission into pose coordination constraints	Simultaneously outputting control variables and pose covariance, with uncertainty directly written into constraints and termination criteria
Digital twins lack decision-level credibility management	Cyber-physical closed-loop assembly [90,91], quality prediction framework [105], multi-physics correction [106]	No assembly system capable of online credibility updating of twin model prediction errors and adaptive control strategy switching based thereon	Twin model calibrated online with measured data, providing confidence bounds and failure trigger conditions for each decision
Unrealized unification of flexible body models and real-time docking planning	Flexible body order reduction and FFRF theory [107,109], shape control and gap optimization [11,12,123]	No reduced-order finite element or FFRF-based flexible body model online embedded into large-scale component contact-aware trajectory planners for synchronous optimization of pose, contact force, and surface error	Planner directly calls flexible models in online solving, real-time correcting paths, loads, and contact sequences
Segmented optimization of gaps, contact forces, shape errors, and compensation processes	Gap-pose optimization [97], coordinated force-shape control [12], single-step shimming [95]	No implementation unifying gap allocation, contact force constraints, shape control, and compensation processes into a single optimization problem	Unified solver simultaneously outputs pose adjustment, actuator loads, and compensation strategies
Industrial verification limited to single-scenario and dedicated test benches	Scaled component verification, single-case engineering verification, partial industrial applications [92,102,104,106]	No unified benchmark verification system across models, environments, and assembly units	Public or semi-public benchmark tasks for unified evaluation of precision, takt time, robustness, and uncertainty indicators
Data-driven methods lack mechanistic constraints and transferability assessment	Sparse learning, machine learning, and reinforcement learning control [93,94,123]	No data-driven closed-loop assembly control framework satisfying physical constraints, assembly safety constraints, and cross-component generalization verification	Data-driven strategies stable across components and boundary conditions, with failure monitoring mechanisms

## Data Availability

The raw data supporting the conclusions of this article will be made available by the authors upon request.
